# A Review of Methods for Predicting Driver Take-Over Time in Conditionally Automated Driving

**DOI:** 10.3390/s25226931

**Published:** 2025-11-13

**Authors:** Haoran Wu, Xun Zhou, Nengchao Lyu, Yugang Wang, Linli Xu, Zhengcai Yang

**Affiliations:** 1 College of Automotive Engineering, Hubei University of Automotive Technology, Shiyan 442002, China; wuhaoran@huat.edu.cn (H.W.); 202311209@huat.edu.cn (X.Z.); xulinli@huat.edu.cn (L.X.); yang516516@163.com (Z.Y.); 2 Key Laboratory of Automotive Power Train and Electronic Control, Hubei University of Automotive Technology, Shiyan 442002, China; 3 Intelligent Transportation Systems Research Center, Wuhan University of Technology, Wuhan 430063, China; lnc@whut.edu.cn

**Keywords:** intelligent transportation, take-over time, driver, prediction mode, influencing factors

## Abstract

Take-over time is a critical factor affecting safety. Accurately predicting the take-over time provides a more reliable basis on issuing take-over requests, assessment of take-over risks, and optimization of human–machine interaction modes. Although there has been substantial research on predicting take-over time, there are still shortcomings in personalized prediction (particularly in accounting for individual differences in driving experience, cognitive abilities, and physiological responses). To gain a comprehensive understanding of the characteristics and applicability of take-over time prediction methods, this review covers four aspects: literature search information, factors influencing take-over time, data acquisition and processing methods, and take-over time prediction methods. Through literature search, research hotspots in recent years have been summarized, revealing the main research directions and trends. Key factors influencing take-over time, including driver factors, autonomous driving systems, and driving environments, are discussed. Data preprocessing stages, including data acquisition and processing, are systematically analyzed. The advantages and disadvantages of classical statistical, machine learning, and cognitive architecture models are summarized, and the shortcomings in current research are highlighted (for instance, the limited generalizability of models trained predominantly on simulator data to real-world driving scenarios). By thoroughly summarizing the strengths and weaknesses of existing research, this review explores under-researched areas and future trends, aiming to provide a solid theoretical foundation and innovative research perspectives for optimizing take-over time prediction, thereby promoting the widespread application and efficient development of autonomous driving technology.

## 1. Introduction

Road congestion and the occurrence of traffic accidents have seen a surge globally. Mitigating these issues has necessitated the development and improvement of autonomous driving technology.

The Society of Automotive Engineers (SAE) standard defines six levels of driving automation (Levels 0–5). In Levels 1–2, the driver remains responsible for vehicle control with automation support for specific tasks. At Level 3 (conditional automation), the vehicle can perform all dynamic driving tasks within its Operational Design Domain (ODD), but requires the driver to resume control when the system encounters scenarios beyond its capabilities. From Levels 3 to 4, the driver progressively cedes physical control in certain situations. Level 5 represents the highest automation, where the vehicle performs all driving functions in all conditions without human input, and typically lacks traditional controls like a steering wheel or pedals [1]. Before achieving fully autonomous driving, a human–machine cooperative driving phase is needed [2,3]. In the case of conditional automation systems, when the system identifies scenarios that fall outside the vehicle’s ODD, the system requests the driver to take over the vehicle through a take-over request (TOR). The driver has to perform the take-over action within a specific time budget (TB).

The take-over process consists of four phases: perception, cognition, decision-making, and response [4]. Take-over time (TOT) refers to the time difference between the TOR notification and the actual take-over action. In the case of the driver’s failure to take-over, the system will initiate risk-mitigation measures to ensure a safe take-over of the vehicle [5]. When a single self-driving vehicle initiates risk-mitigation measures, the effect of the measures does not significantly influence the traffic system. However, when multiple self-driving vehicles concurrently implement risk-mitigation measures, the measures can cause traffic congestion or accidents [6]. Determining the take-over time and improving the efficiency of the take-over process to increase the success rates of take-over actions without hampering the driver during the take-over process are important for the safe take-over of the vehicle.

Despite the extensive coverage of TOT prediction methods in existing literary works, gaps and inadequacies remain. Existing literary works predominantly involve the analysis of singular factors instead of jointly taking the impact of multiple factors into account for the prediction of TOT. Also, a majority of studies have been conducted based on the simulator data instead of gaining sufficient exposure to real-world cases of driving.

**Figure 1:**
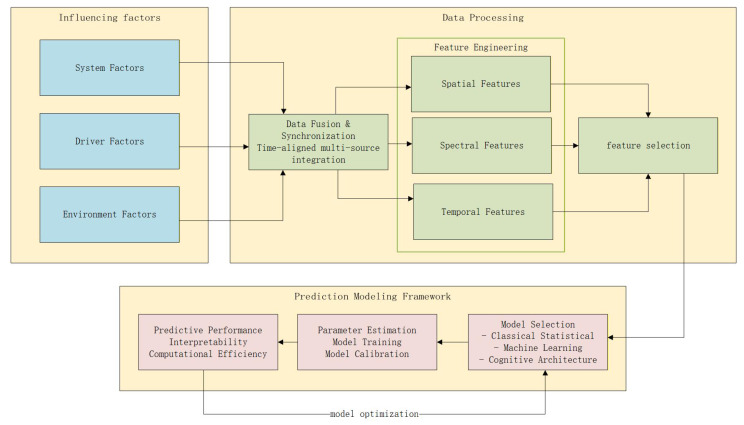
A Framework for Take-over Time Prediction Based on Multi-Modal Data Fusion.

To systematically review the state-of-the-art in TOT prediction and address the identified research gaps, this paper conducts a comprehensive survey structured around three core research questions (RQs). The overarching goal is to move beyond isolated analyses and present a synthesized view of the TOT prediction pipeline. To visually articulate this integrative approach, Figure 1 presents a unified modeling framework that captures the interplay between key components, from data acquisition to model optimization. This review is guided by the following RQs:

- RQ1: What are the key factors that influence take-over time, and how do they interact? This question aims to synthesize the multifaceted influences on TOT—encompassing driver states, environmental conditions, and TOR characteristics—to advance beyond analyses of factors in isolation.
- RQ2: What are the primary methods for data collection and processing in TOT research, and what are their respective challenges?This question seeks to critically outline and compare experimental paradigms, data acquisition techniques, and preprocessing methods, with a particular focus on the gap between simulator-based and real-world data.
- RQ3: What are the prevailing methodological approaches for predicting TOT, and how do their performance and applicability compare across different scenarios? This question focuses on reviewing, classifying, and evaluating the prediction models themselves, ranging from statistical analyses to machine learning techniques, to elucidate their strengths and limitations.

By addressing these questions within the structured framework of Figure 1, this study not only summarizes the existing research landscape but also provides a coherent foundation and clear guidance for future work, ultimately aiming to enhance the safety and effectiveness of autonomous driving systems.

## 2. Literature Search Information

To understand the development trends in research on TOT prediction, this paper conducted a comprehensive literature search across multiple major academic databases, including Web of Science Core Collection, Scopus, IEEE Xplore, and Engineering Village (Compendex). The search utilized keywords such as ‘take-over time prediction model’, ‘take-over time prediction’, ‘take-over time’, ‘performance prediction’, and related terms to retrieve relevant scholarly publications from 2016 to 2024. The selection criteria included the field of autonomous driving, take-over experiments, and the analysis of TOT data. A total of 143 papers were selected. Using the BiblioShiny package in R [7] and other visualization tools, the analysis covered four aspects: literature type, keywords, countries and regions, institutions, and journals.

### 2.1. Literature Type

**Figure 2:**
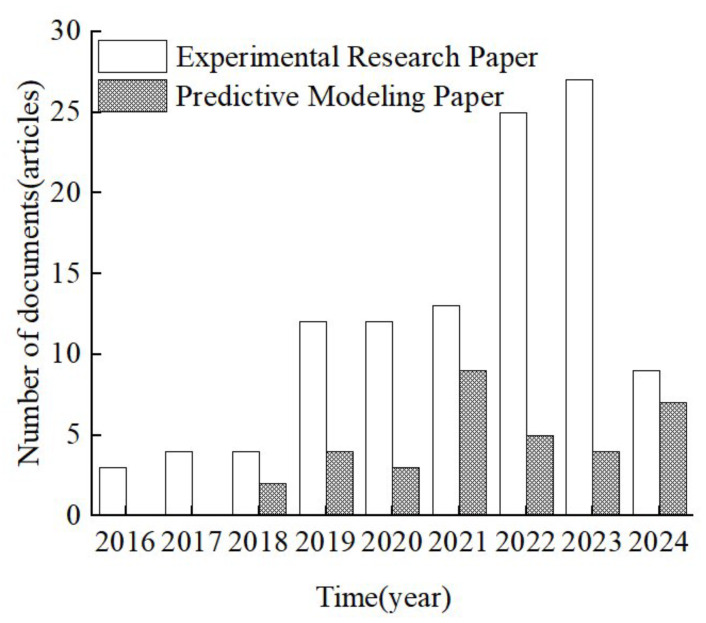
Number of Experimental and Predictive Model Publications.

This paper categorizes the selected literature into two types: papers that conduct take-over experiments, analyzing the influencing factors of TOT and papers that establish TOT prediction models based on take-over experiments. As shown in Figure 2, from 2016 to 2018, the amount of literature on both categories was relatively small. After 2019, the number of experimental papers increased significantly, peaking in 2023 with 27 papers. The number of prediction model papers has shown a growing trend, but with a relatively smaller increase, averaging around 6 papers per year.

### 2.2. Keywords of the Literature

**Figure 3:**
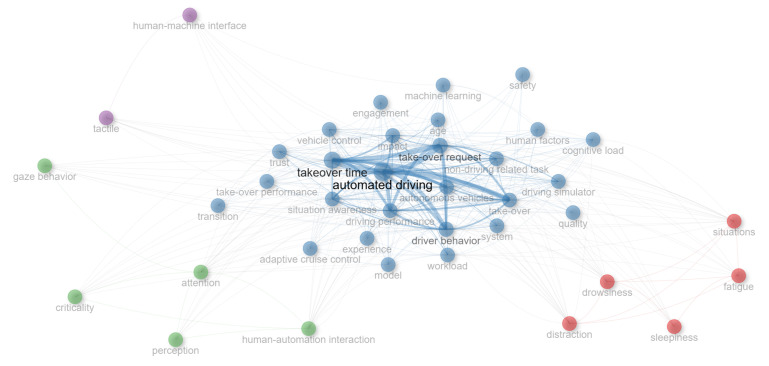
Co-occurrence Network of Keywords.

Figure 3 presents a keyword co-occurrence network generated using the bibliometrix package in R, which visually delineates the intellectual structure of the research field based on the 39 most frequent keywords. In the network, node size corresponds to keyword frequency, and the thickness of the connecting curves indicates the strength of co-occurrence between terms. Keyword clusters were identified using the Walktrap algorithm, which detects communities in the network based on short random walks. The most prominent feature of the map is a large, densely interconnected blue cluster positioned centrally, which unequivocally represents the core research theme. This cluster is anchored by the central and most frequent term ‘automated driving’, which exhibits the strongest linkages to pivotal concepts such as ‘take-over time’, ‘take-over request’, and ‘driver behavior’. The high frequency and tight cohesion of these keywords indicate that the primary research focus is concentrated on the dynamics of control transitions and driver performance within autonomous vehicles. Surrounding this central core, several smaller clusters in distinct colors represent specialized sub-themes that interact with the main research stream. The red cluster, grouping keywords like ‘distraction’, ‘drowsiness’, and ‘fatigue’, highlights the significant sub-topic of “Driver State and Impairment”. Adjacent to the core, the green cluster, encompassing ‘situation awareness’, ‘attention’, and ‘trust’, can be interpreted as the “Human Cognition and Reliability” theme. Other clusters, such as the one containing ‘human–machine interface’ and ‘tactile’, signify more niche research areas concerning interaction modalities. The structural centrality of the blue cluster, coupled with the radial distribution of secondary themes, effectively illustrates that investigations into vehicle control transitions form the foundational axis from which other specialized inquiries branch and connect.

### 2.3. Countries and Regions

**Figure 4:**
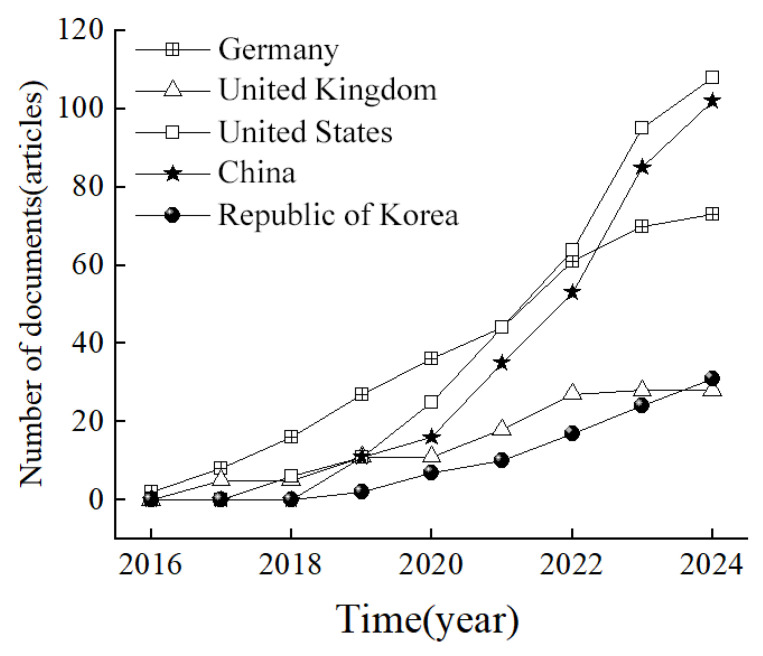
Cumulative Literature from Various Countries.

Figure 4 shows the dispersion of bibliography among the top 5 countries with the highest publication volumes. These statistics are based on the countries where the authors’ institutions are located. A single article may therefore have authors from different countries. The top three countries in terms of publication volume are the United States (108), China (102), and Germany (73), followed by Republic of Korea (31) and the United Kingdom (28). Both China and the United States have shown rapid growth in annual publication volumes in this field, while Germany and the United Kingdom have maintained stable publication quantities. These data indirectly reflect the continuous attention and proactive investment of China and the United States in research.

Table 1 presents the top 10 countries in terms of total citation frequency, while Table 2 showcases the top 10 countries based on average citation frequency. The average citation frequency is calculated as the ratio of total citations to the number of published articles from each country. Germany leads with the highest total citation frequency (901), followed by the United Kingdom (461), The Netherlands (345), and the United States (308). In terms of average citation frequency, Australia tops the list (109.5), followed by the Netherlands (57.5), United Kingdom (51.2), and Germany (42.90). Although the United States and China have advantages in publication volume, their average citation frequencies are relatively low. This suggests a high level of attention in this field from both countries, but the difference in citation frequencies reflects variations in research quality and impact. Despite having lower publication volumes compared to China and the United States, Germany and the United Kingdom demonstrate higher quality literature and exhibit strong academic influence. These data provide valuable insights for evaluating the influence and development potential of various countries in the field of autonomous driving research.

### 2.4. Institutions and Journals

Institutions with the highest publication volume are shown in Table 3. The University of Michigan leads with the highest number of publications, reaching 19 articles, followed by Tsinghua University with 15 articles. Beihang University, Delft University of Technology, and two other institutions each contributed 11 articles, while the remaining institutions had publication volumes ranging from 8 to 9 articles. The research achievements of these universities have played a significant role in driving the development of the take-over field.

Journals with the highest number of publications in this field are presented in Table 4. Among them, “Transportation Research Part F: Traffic Psychology and Behaviour” ranks first with the highest publication volume, reaching 21 articles. “Accident Analysis and Prevention” and “Human Factors” also stand out with 18 and 15 articles respectively. Other significant journals include “IEEE Transactions on Intelligent Transportation Systems” and “Applied Ergonomics”. These journals serve as the primary platforms for publishing take-over research, reflecting the latest advancements.

## 3. Factors Affecting Take-Over Time

The driver, the automated driving system, and the driving environment are the three core elements that influence vehicle safety and comfort in take-over scenarios [5]. The interaction among these three factors jointly determines the behavior and performance during the take-over process. When analyzing the factors affecting TOT, a comprehensive consideration of the impacts from these three aspects should be taken into account.

### 3.1. Driver Factors

Conditional automated driving allows the driver to be free from the continuous burden of driving tasks, providing opportunities for them to engage in non-driving related tasks (NDRTs). Drivers may experience varying degrees of distraction, concentration, excitement, and fatigue due to the influence of NDRT. Generally, engaging in NDRT tends to increase the TOT [8]. Considering the existence of individual differences, even if performing the same NDRT, the TOT may vary. Therefore, when analyzing driver-related factors that affect TOT, two aspects can be explored: one is the state of engaging in NDRT, and the other is the individual driver differences.

#### 3.1.1. State of Engaging in Non-Driving Related Tasks

Regarding the influence of the state of engaging in NDRT on the driver’s TOT, the academic community has not yet reached a consensus and still remains somewhat controversial. The types of NDRTs in typical take-over experiments are detailed in Table 5. Generally, the more complex the NDRT, the longer the driver’s TOT [9]. For example, tasks like making phone call and answering questions require the subjects to fully utilize their senses and have a higher demand for memory. Certain tasks (2-back and SuRT) further necessitate manual manipulation and verbal responses, leading to extended TOT. In comparison, tasks like watching videos, resting with eyes closed, or reading are simpler tasks that demand less of the senses and have a lower demand for memory, and in most cases, do not require manual manipulation or responses, thus having a smaller impact on TOT. According to the task load level, NDRTs can be classified into low-load and high-load tasks [10]. High-load NDRTs are usually complex and result in longer TOT [11]. However, subjects who are very familiar with specific complex NDRTs may consider them as low-load, such as counting change or search tasks.

Based on the correlation between task involvement and fatigue (or distraction) driving, NDRTs can be categorized into those that cause fatigue (or distraction) and those that counteract fatigue (or distraction). If the subject is already in a fatigued state (long-term rest with closed eyes) before the take-over, moderately engaging in NDRTs can help alleviate fatigue [12]. Prolonged passive observation of the environment can lead to distraction and extend the TOT, while moderately engaging in other NDRTs can invigorate the subject, alleviate distraction, and bring the TOT back to normal [13]. Even with the same NDRTs (read magazine, listen to music, and read book), differences in engagement level may significantly affect the length of the TOT [14].

The above analysis results indicate that NDRT have a significant impact on TOT in terms of complexity, load level, the subject’s state before take-over, and engagement level. Therefore, when evaluating the take-over process of an automated driving system, it is essential to fully consider the specific characteristics and requirements of NDRTs to better understand their impact on TOT. This understanding can help optimize the driver’s take-over experience and ensure road safety.

**Table 5:** Typical Non-Driving Related Tasks in Take-over Experiments.

Non-Driving Related Tasks	Sensory	Movement	Language	Memory	Ref.
Observe Surrounding Environment	Visual	N/A	F	T	[9,15,16,17]
Watch Video	Visual, Audio	N/A	F	N/A	[14,15,18]
Make Phone Call	Audio	T	T	T	[9]
Have a Conversation	Audio	N/A	T	T	[9,14]
Answer Questions	Visual, Audio	F	T	T	[14,19]
Read Book	Visual	N/A	F	N/A	[9]
Listen to Music	Audio	F	F	N/A	[14]
Listen to Audiobook	Audio	N/A	F	T	[16]
Read Magazine	Visual	T	F	N/A	[14,16]
2-Back (Visual)	Visual	T	F	T	[20]
2-Back (Audio)	Audio	F	T	T	[21]
Rest with Eyes Closed	N/A	F	F	F	[9]
Send Text Messages	Visual	T	F	T	[9]
Count Change	Visual	T	F	T	[9]
Search Task	Visual	T	F	T	[16]
SuRT	Visual	T	F	T	[10,20,22,23,24]
Play Tetris	Visual	T	F	T	[16]
Play 2048	Visual	T	F	T	[25,26]

#### 3.1.2. Individual Differences

Multiple studies have explored the influence of individual differences on TOT from various perspectives. Common research areas include age [27], gender [28], psychological and physiological states [3,25,29], and autonomous driving experience [28,30,31,32,33]. Generally, older individuals have longer TOT compared to younger ones [27]. Muslim et al. [28] argue that gender has no significant effect on TOT, while Li et al. [34] suggest that women have shorter TOT.

Subjects exhibit significant differences in TOT under different psychological and physiological conditions. For instance, Sanghavi et al. [25] found that drivers under anger had longer TOT, as the anger state causes distraction and diminished judgment, adversely affecting take-over performance. On the other hand, Becker et al. [29] discovered that subjects with lower trust in the system were more prone to disengage from autonomous driving. Additionally, Huang et al. [23] indicated that extroverted drivers have relatively shorter TOT.

Research shows that less autonomous driving experience results in longer TOT [28]. To mitigate potential learning effects, the order of take-over experiments is often adjusted [17]. With the accumulation of autonomous driving experience, subjects’ operational proficiency gradually improves, resulting in shorter TOT [30,31,32]. Rydström et al. [33] demonstrated that even novice drivers can maintain TOT within safe limits through short-term training. Furthermore, Roberts et al. [35] showed that the 3M training program (including Mistakes, Mentoring, and Mastery) effectively improves drivers’ takeo-ver performance.

Existing research on the effects of age, gender, and psychological and physiological states on TOT varies, possibly due to differences in study samples and methodologies. The specific mechanisms of these influences require further investigation. In contrast, the improvement effect of autonomous driving experience on TOT is relatively clear; the more experience, the shorter the TOT. Therefore, exploring and developing effective training methods is of significant research and practical value for enhancing subjects’ take-over abilities. This not only helps improve drivers’ operational proficiency but also enhances overall road safety.

### 3.2. Autonomous Driving System

The impact of autonomous driving systems on TOT involves numerous factors. Currently, research primarily focuses on three key aspects: TOR, TB, and take-over method. The TOR is a critical signal sent by the autonomous driving system to the driver, and its design, timing, and information clarity directly affect the driver’s TOT. The TB, which is the TOT allocated to the driver, is crucial for ensuring the smooth process of take-over. A reasonable TB ensures that the driver can intervene promptly when the system requires it, thereby ensuring road safety. The take-over method also significantly influences TOT. Therefore, when exploring the impact of autonomous driving systems on TOT, it is essential to fully consider the role of these three key factors.

#### 3.2.1. Take-Over Request

An in-depth analysis reveals that TOR design is the core factor influencing TOT. As detailed in Table 6, TOR modalities are primarily categorized as unimodal (e.g., visual or auditory signals) or multimodal (e.g., audiovisual or audiovisual-tactile combinations) [36].

Visual signals provide rich information, allowing subjects to quickly adapt to the current driving environment [37]. If NDRTs occupy visual resources, the effectiveness of visual signals will be limited. Normally, visual signals work in concert with other signals to exert their influence collectively.

Auditory signals can be divided into language-based and non-language-based signals. Language-based signals offer strong explanatory power but require some time for comprehension. Non-language-based signals, such as white noise and pure music, allow drivers to understand faster, but their intense or rhythmically strong nature may lead to attention distraction. Additionally, an overload of warning signals in the system can add to the memory burden from non-language-based signals, potentially causing memory confusion.

Tactile signals are typically delivered through the seat or the seatbelt, providing intuitive and immediate warnings [38]. If there are too many types of tactile signals, they can similarly increase the memory burden on the driver.

Yun and Yang [39] believe that audiovisual-tactile signals are the most effective, with pure visual signals being the least effective. Laakmann et al. [38] found that audiovisual-tactile signals can significantly shorten TOT. However, Lee et al. [40] argue that audiovisual signals are more effective. Additionally, Ko et al. [26] discovered that the effectiveness of TOR is influenced by individual differences, leading to different outcomes.

To enhance the driver’s experience, design of TOR should focus on precision, speed, comfort, minimal memory burden, and personalization. This approach aims to optimize system performance and avoid potential issues arising from information overload or discomfort.

**Table 6:** Typical Take-over Requests in Take-over Experiments.

Take-Over Request	Specific Content	Ref.
Auditory	Audio Alarm	[41]
	Beeper	[22]
	Beep Sound	[14,42,43]
	Mixed Frequency Alert Tone	[39]
Visual	Display Icon and Steering Wheel LED Flashing	[44]
	Flashing Red Image	[39]
	Changing Color Lighting	[45]
Tactile	Seatbelt and Seat Vibration	[46]
	Seat Vibration	[47]
	Bottom Seat Vibration	[39]
Visual + Auditory	Screen Icon + Mixed Frequency Alert Tone	[48]
	Screen Text and Ambient Light + Bell/Beep Sound	[40]
	Beep Sound + Red Text Image	[42]
	Screen Icon + Buzz Sound	[18]
	Visual Cue + Female Voice	[43]
	Screen Image + Standard Warning Tone (Beep)	[38]
	Display Text + Non-verbal Alert Sound	[49]
Visual + Tactile	Flashing Red Image + Bottom Seat Vibration	[39]
	Changing Color Lighting + Seat Vibration	[45]
	Screen Image + Seatbelt Vibration	[38]
Auditory + Tactile	Mixed Frequency Alert Tone + Bottom Seat Vibration	[39]
Visual + Auditory + Tactile	Flashing Red Image + Mixed Frequency Alert Tone + Bottom Seat Vibration	[39]
	Bar LED + Boeing 747 Alarm Sound + Backrest Vibration	[50]
	Screen Image + Standard Warning Tone + Seatbelt Vibration	[38]

#### 3.2.2. Time Budget

**Figure 5:**
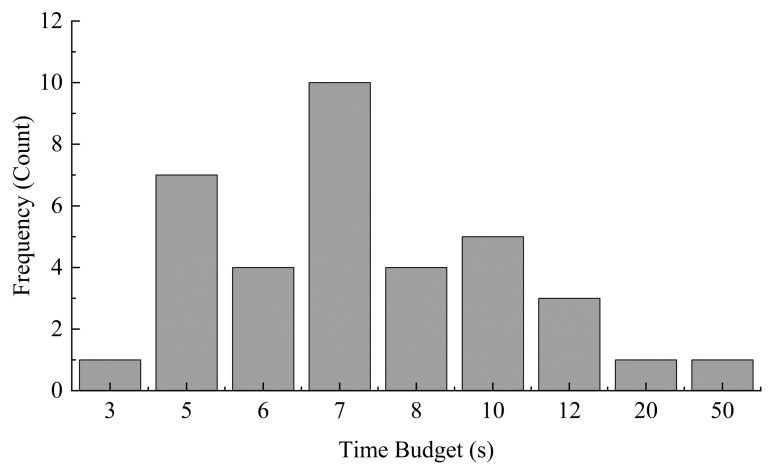
Distribution of Time Budget Settings in take-over Studies.

When analyzing factors influencing TOT in autonomous driving systems, TB is an essential component and cannot be overlooked. TB not only significantly affects the driver’s TOT but also directly determines the overall safety of the take-over process. According to literature statistics over the past three years, the TB situation is shown in Figure 5. Some studies have explored various TB and provided detailed analysis of driver performance under each budget condition. The effects of different TB on TOT can be summarized as follows: firstly, excessively long TB can reduce the driver’s trust in autonomous driving systems [51], thereby affecting the driving experience; secondly, within the premise of ensuring safety, appropriately shortening the TB can help improve road traffic efficiency; however, if the driver’s take-over is too fast, it may lead to insufficient preparation, thereby affecting the stability of manual driving [31,52]. Therefore, a balance point should be sought between improving traffic efficiency and ensuring safe take-over.

According to previous studies [53], within a certain time range, a longer TB can enhance take-over safety. Conversely, an overly short TB increases the subject’s workload, thus increasing danger, which aligns with the viewpoints of other studies mentioned above.

#### 3.2.3. Take-Over Methods

The choice of take-over methods is of great significance in the design of autonomous driving systems, as it directly relates to the safety of the driver’s take-over and the overall riding experience. As shown in Table 7, take-over methods include steering, pedal pressing, and button pressing. Some studies may employ a combination of multiple take-over methods to enhance flexibility. However, in specific take-over events, the choice of take-over method may be restricted; for example, in highway scenarios, pedal take-over might be more relied upon. Additionally, a few studies innovatively introduced custom take-over methods, such as pressing a lever on the back of the steering wheel or touching the steering wheel while simultaneously pressing a button, to meet specific experimental needs.

In the design process of take-over methods, several aspects can be considered. Firstly, allowing multiple take-over methods may better accommodate individual differences among drivers. Secondly, to ensure driving safety, it is necessary to appropriately restrict take-over methods based on different take-over events. Lastly, exploring and designing diverse take-over methods can help discover better solutions, thereby enhancing overall system safety and comfort.

### 3.3. Driving Environment

In exploring factors related to TOT, the driving environment is an important dimension that cannot be ignored. The impact of the driving environment on TOT can be analyzed from two perspectives: environmental factors and take-over events. Environmental factors such as adverse weather conditions and complex traffic situations not only affect the driver experience but may also weaken the system’s ability to recognize and process environmental information. When the system fails or cannot accurately recognize and process environmental information, it may trigger a take-over event. Comprehensive consideration of these factors can help to more fully evaluate the performance of take-over events in experiments.

#### 3.3.1. Environmental Factors

**Figure 6:**
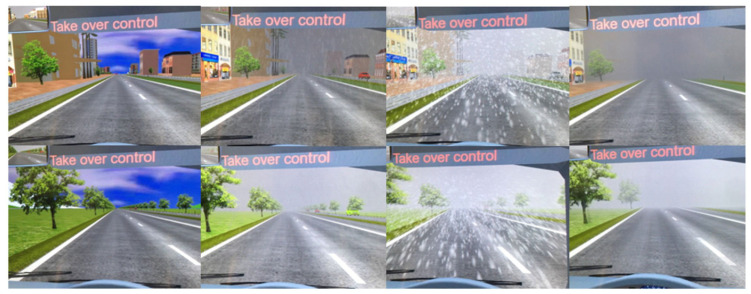
Sunny, rainy, snowy, and foggy conditions [66].

When analyzing the impact of environmental factors on TOT, weather conditions are a significant aspect. Changes in weather conditions directly affect driving safety and driver performance. As shown in Figure 6, common weather conditions include daytime, nighttime, sunny, rainy, foggy, and snowy conditions [64]. Daytime and sunny weather provide clear visibility, enhancing driving safety. However, at night, due to inadequate lighting, drivers need to spend more time and effort adapting to the environment, which not only increases visual burden but may also prolong TOT [65]. Rainy and snowy conditions significantly reduce road friction coefficients, increasing the risk of sliding and loss of control. Foggy conditions significantly reduce visibility, affecting the system’s environmental perception capabilities and causing driver fatigue and anxiety. Adverse weather conditions not only increase the burden on the system but also lead to extended cognitive decision-making time for drivers, thereby increasing TOT.

The impact of traffic situations on take-over safety has received widespread attention. Li and Xuan [67] argue that the higher the traffic flow density, the greater the risk of take-over safety. Yang et al. [42] suggest that the more complex the road environment and the higher the traffic flow, the lower the safety of take-over. Wang et al. [41] found that a higher relative speed leads to a longer TOT. Additionally, Scharfe-Scherf and Russwinkel [68] believe that the driver’s familiarity with traffic situations and the complexity of the environment significantly affect subjective complexity and TOT. It is worth noting that autonomous driving systems typically come equipped with various sensing devices, such as LiDAR, cameras, and millimeter-wave radars. If these devices can provide detailed information when the driver needs to take-over, it will help the driver gain a deeper understanding and adapt to the current traffic situations.

#### 3.3.2. Take-Over Events

Take-over events are closely related to environmental factors, and a deeper exploration of these events is crucial for understanding take-over safety. As shown in Table 8, common take-over events can be mainly categorized into three types. The first type is restricted longitudinal functionality of the system, which includes encountering stationary obstacles, dynamic obstacles, or adverse weather conditions. These situations may hinder the system’s ability to accurately handle acceleration, deceleration, or braking operations, affecting longitudinal control of the vehicle. In the face of oncoming obstacles, drivers can slow down, stop, or steer to avoid them; when encountering adverse weather or steep slopes, drivers should slow down or stop to ensure safety. The second type is restricted lateral functionality of the system, which includes scenarios such as unclear lane markings and ramp entrances/exits. When lane lines are unclear, the system struggles to accurately identify lane boundaries, impacting lane-changing operations and stable driving within the lane; when approaching a ramp entrance/exit, due to the complex road structure, the system struggles to handle lane changes; in such situations, drivers should promptly take-over control to ensure safe vehicle operation. The last type is system failure, which occurs when some or all system functions fail, causing the system to malfunction and preventing normal operation. Drivers should promptly identify the system issues and immediately take-over to ensure safe driving.

In summary, there has been a significant amount of research focused on uncovering the influencing factors and patterns of TOT. However, such studies have not yet fully aligned with the complexity requirements of autonomous driving technology, and many details and special cases still need in-depth exploration. Additionally, the interrelationships among various influencing factors remain unclear. Clarifying these relationships will help to more accurately explain the differences in experimental results, thereby improving the precision of TOT predictions.

## 4. Data Acquisition and Processing Method

Data acquisition and processing are the cornerstones of ensuring the accuracy and reliability of research results. This paper will detail how, during the experimental research process, a series of systematic and standardized methods are used to acquire and process data, ensuring the authenticity and scientific value of the final analysis results. The following sections will respectively elaborate on the use of various high-precision acquisition devices, the specific processes of data collection, key methods of feature extraction, and rigorous criteria for data screening, laying a solid foundation for predicting TOT.

### 4.1. Data Acquisition

For most researchers, valuable data primarily focus on the seconds to minutes before and after the take-over, while few researchers pay attention to the entire experimental process. Data acquisition devices can be divided into two categories: one is simulator equipment, which includes built-in vehicle sensors, mainly used to collect information about the surrounding environment, such as vehicle speed, acceleration, steering, etc. The other category is external devices, such as cameras, eye trackers, electro-signal patches, infrared sensors, etc., which can record physiological and behavioral data of subjects in real-time, including eye movement trajectories, physiological signals, body surface temperature, etc.

As shown in Table 9, experimental equipment can be roughly classified into three types [4]: desktop simulators, cockpit simulators, and actual vehicles. Most studies use simulators for experiments due to their advantages in safety, cost-effectiveness, and the ability to simulate diverse and critical scenarios. However, a key challenge with simulators is ensuring ecological validity, as they cannot fully replicate the physical and psychological pressures of real-world driving, potentially leading to data that misses subtle practical issues. In contrast, actual vehicle experiments provide high-fidelity data and a genuine driving experience. Yet, they introduce significant challenges, including substantial safety risks, high costs, and the practical difficulty of robustly deploying multiple data acquisition devices in a moving vehicle without interfering with the driver. Additionally, Pipkorn et al. [78] used the Wizard of Oz (Woz) method for experiments. In this method, the vehicle is equipped with two drivers: one is the subject, and the other acts as the operator of the autonomous driving system. The operator controls the vehicle according to the experimental requirements to simulate the autonomous driving state. This method lowers experimental costs through manual control, allowing researchers to flexibly adjust experimental conditions. A primary limitation of this approach, however, is that manual control cannot fully reproduce the precise timing and behavioral nuances of a real automated system, which may affect the generalizability of the findings.

**Figure 7:**
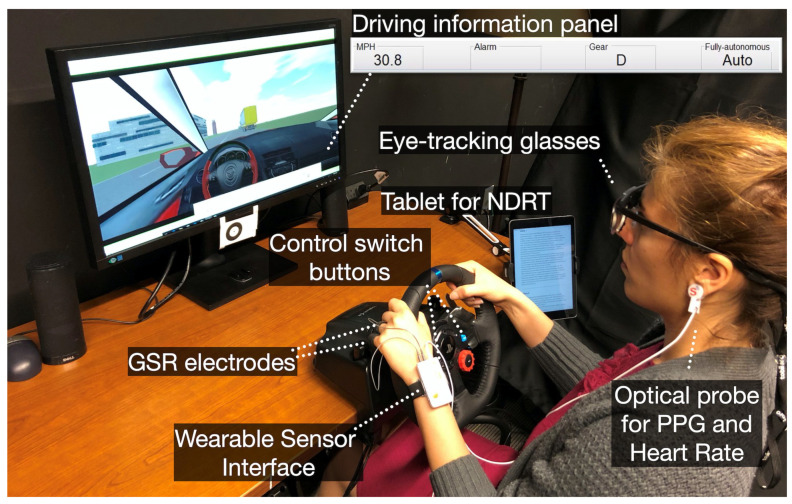
Typical Data Collection Equipment in Take-over Experiments [63].

Common data acquisition devices are shown in Figure 7. An eye tracker is used to track the subject’s eye movements; electro-signal patches monitor physiological signals; cameras record posture and actions; and infrared sensors measure body surface temperature. A major technical challenge in employing these devices, especially in combination, is achieving precise temporal synchronization across all data streams (e.g., aligning a gaze point with a specific vehicle event). Furthermore, data quality from physiological sensors and cameras is highly susceptible to artifacts caused by participant movement, changes in ambient lighting, and equipment limitations, requiring sophisticated post-processing for signal cleaning.

Table 10 lists common data types, which not only include psychological and physiological indicators but also reflect various information during the take-over process. Visual and facial data can reveal the driver’s attention distribution and changes in expression. Du et al. [56] consider frames with eye movement speed below $100^{{}^{\circ}}$/s as fixation. Experimental type data covers factors such as age, gender, NDRTs, and take-over methods; psychological evaluation data can assess the driver’s trust, anger, distraction, and fatigue; physiological data records the driver’s heart rate, skin conductance, respiration, and body temperature; and vehicle data provides detailed information about the vehicle’s position, speed, acceleration, and turning angle. Multi-dimensional data provide a solid foundation for analysis, but their richness also presents the challenge of integrating and interpreting disparate data modalities (e.g., correlating a physiological stress response with a specific driving maneuver) to form a coherent model of driver state and performance.

If the acquisition equipment is insufficient, data can be obtained from open-source databases such as the 100-Car Naturalistic Driving Study [85], SHRP 2 Naturalistic Driving Study [86], and the Shanghai Naturalistic Driving Study [87]. While invaluable, leveraging these datasets presents its own set of challenges, such as potential mismatches in data formats or recorded variables with the specific research questions of a take-over study, limiting the flexibility of secondary analysis.

**Table 10:** Data Types Collected in Take-over Experiments.

Data Type	Specific Content	Ref.
Visual Data	Gaze	[14,16,56,58,63,88]
	Saccade	[56,58]
	Pupil area	[56,58,63]
	Blinking	[56]
	Facial direction	[48]
	Head posture	[56,89]
Experiment Type Data	Age/Gender/NDRTs/Take-over mode	[90]
Psychometric Data	Drowsiness	[16]
	NDRTs engagement	[14]
	Distraction score	[18]
	Risky driving tendency	[83]
	System trust	[26]
Physiological Data	Respiration	[59]
	Heart rate	[22,56,63]
	Skin conductance response	[22,56,63]
	Electrocardiography	[48]
	Electroencephalography	[91]
Limb Data	Hand position	[14,48,88,90]
	Foot position	[48,88,90]
	Body posture	[48]
Vehicle Data	Position/Speed/Acceleration/Steering angle	[19,41,63]

### 4.2. Data Processing

In take-over research, accurate data processing is a critical step to ensure the reliability and validity of the results. Different types of data require specific processing methods to ensure their accuracy and usability. This article will detail the processing methods for survey questionnaire data, video data, Electrocardiography (ECG) data, and general data processing techniques, helping researchers better understand and apply these technologies.

In psychological, human factors, and social science research, survey questionnaires are a common data collection tool used to assess the psychological state, attitudes, and behavioral characteristics of subjects [92]. Using different types of scales, researchers can collect rich data for in-depth analysis and interpretation. Scales such as the 7-point Likert scale [16], 10-point Likert scale [18], 11-point Likert scale [73], NASA Task Load Index (NASA-TLX) [14,26,83], and the 10-item Perceived Stress Scale (PSS-10) [63] are widely used to assess key indicators such as distraction scores [18] and drowsiness scores [85]. Additionally, Yi et al. [93] innovatively introduced machine learning models to evaluate changes in trust during the subject’s take-over process. Dogan et al. [94] used the Local Outlier Factor (LOF) method to detect the state of subjects before take-over. Teshima et al. [48] utilized convolutional neural networks to identify the state of subjects.

Video data is commonly used to record and analyze subjects’ behavioral patterns and attention distribution. By coding video data, researchers can extract key information about subjects’ gaze behavior, head posture, and body movements. For example, the number and duration of gaze fixations on different locations [14], gaze direction, and take-over processes [88]. Yi et al. [22] defined two areas of interest (AOI) and recorded subjects’ gaze durations. Wu et al. [58] utilized a modified driver monitoring system (DMS) to record data such as saccades, pupil size, number of fixations, and gaze durations. Du et al. [56] used Smart Eye to record subjects’ head posture, blinking, fixation duration on AOIs, and saccade counts in real-time. Yoon et al. [14] employed an eye tracker to document subjects’ attention distribution. Pakdamanian et al. [63] extracted features from raw data using imotion software, including gaze position, pupil size variation, fixation duration, and gaze sequence. To minimize distractions caused by wearable eye trackers, Lotz and Weissenberger [89] collected visual and head position data using a non-invasive eye tracker called Smart Eye Pro, along with Microsoft Kinect to record subjects’ body posture. Additionally, Berghöfer et al. [16] extracted visual features using hierarchical clustering. Li et al. [95] assessed driver fatigue by combining the Percent of Eyelid Closure at 80% (P80) with eye movement data measured every 60 s. Araluce et al. [96] tracked subjects’ areas of focus by combining gaze fixation points with semantic segmentation.

For physiological signals such as respiration and ECG signals, researchers typically remove noise and extract key information. Yi et al. [22] filtered the galvanic skin response (GSR) signals to remove noise and motion artifacts, extracting the rapid phase component of GSR; they used a QRS detection algorithm to extract R-peaks from ECG signals, obtaining the normal-to-normal heartbeat intervals (RR) and replacing outliers with mean values; they employed a self-threshold detection method to obtain heart rate from ECG signals; additionally, they used min-max normalization to eliminate individual differences in GSR, HR, and RR intervals; they standardized individual differences by dividing the phase component of GSR by the subject’s maximum value. Du et al. [56] used the Shimmer3 GSR+ device, including GSR electrodes and photoplethysmography (PPG) probes, to collect GSR and HR data, and implemented real-time synchronization and visualization of data through imotion software. They also used min-max normalization for the subjects’ feature values. Pakdamanian et al. [63] measured skin conductivity and heart rate using GSR and PPG sensors embedded in a smartwatch to monitor subjects’ stress levels. Unlike some heart rate monitoring devices that rely on metal electrodes placed on the chest, this method does not require invasive physical contact. The PPG sensor monitors heart rate by emitting infrared light into the body and estimating blood flow by measuring reflected light. Pakdamanian et al. [63] normalized the raw PPG signals using min-max normalization and used the open-source toolkit HeartPy to filter the PPG signals, extracting the following features from heart rate variability (HRV) analysis: the standard deviation of normal heartbeats (SDNN), the root mean square of successive differences in normal heartbeats (RMSSD), and the proportion of successive heartbeat pairs with differences greater than 50ms (pNN50). They also obtained two important GSR features: the number of peaks and their amplitudes.

Different types of data often face issues with missing values or require standardization during the collection and processing phases. To effectively reduce the impact of missing values, Ayoub et al. [97] constructed missing values as dummy variables to minimize their influence. Kim et al. [83] treated outliers by removing values exceeding three times the standard deviation. Gruden and Jakus [98] performed z-normalization on the data. Liu et al. [99] used min-max normalization on the data.

Researchers typically perform correlation analysis on the processed data against the defined TOT to extract highly correlated features. Wu et al. [58] calculated the Pearson correlation coefficient to analyze the correlation between independent variables and TOT, and used stepwise regression (backward elimination) to screen out the predictors that significantly affect TOT. Lotz and Weissenberger [89] used multivariate analysis of variance (MANOVA) to screen independent variables. Yoon et al. [14] analyzed the variance inflation factor (VIF) to avoid high correlation among predictors. Pakdamanian et al. [63] obtained a stable and independent subset of features using the least absolute shrinkage and selection operator (LASSO) and ranked the features using a random forest.

Researchers typically perform correlation analysis on the processed data against the defined TOT to extract highly correlated features. The selection of specific methodologies is guided by their underlying statistical principles and the nature of the data. Wu et al. [58] calculated the Pearson correlation coefficient, which operates on the principle of measuring the linear covariance between two variables normalized by their standard deviations, providing a dimensionless index between −1 and 1. They further used stepwise regression, whose rationale lies in an iterative algorithm that adds or removes features based on hypothesis testing (e.g., *p*-values) of the estimated coefficients, seeking to optimize a model fitness criterion like AIC or BIC. Lotz and Weissenberger [89] used multivariate analysis of variance (MANOVA), a method whose rationale extends from ANOVA by evaluating the combined variance of multiple correlated dependent variables, using test statistics like Wilks’ lambda to protect against Type I errors that might occur when running separate ANOVAs. Yoon et al. [14] analyzed the VIF, a principle based on the coefficient of determination (R^2^) obtained by regressing a predictor against all other predictors. A high VIF (typically > 5 or 10) indicates multicollinearity because it signifies that the variance of the coefficient estimate is inflated due to this redundancy. Pakdamanian et al. [63] used the least absolute shrinkage and selection operator (LASSO), a principle that introduces an L1-norm penalty term to the regression loss function. This penalty has the geometric effect of shrinking some coefficients to exactly zero, performing continuous feature selection. They then used a random forest for feature ranking, which is based on the principle of measuring the average decrease in node impurity (e.g., Gini index or entropy) across all trees in the ensemble when a feature is used for splitting, or by permuting features and measuring the resulting increase in prediction error.

## 5. Take-Over Time Prediction Methods

The purpose of predicting drivers’ TOT is to comprehensively analyze the impact of various factors on TOT and make predictions based on this analysis. Examining the influence of a single factor or the independent impact of each factor is not comprehensive enough and is difficult to accurately reflect the overall situation. To improve prediction accuracy, multiple dimensions of variables are usually used as model inputs, enabling the model to integrate various information and more comprehensively consider all influencing factors, thereby potentially providing more accurate predictions of drivers’ TOT. TOT models generally fall into three categories: classical statistical models, machine learning models, and cognitive architecture models.

### 5.1. Classical Statistical Models

In research on TOT prediction, classical statistical models are widely adopted due to their transparency, interpretability, and mature technical foundation. These models can handle various data types and have demonstrated strong predictive performance in numerous studies. For instance, commonly used approaches include multiple regression model, linear mixed-effects model, multiple linear regression model, generalized non-linear regression model, generalized additive model, and generalized linear mixed model. A summary of research on classical statistical models for TOT prediction is provided in Table 11.

Research on TOT prediction frequently employs classical statistical models due to their interpretability and well-established theoretical foundation. A comparative analysis of the methodological approaches (Table 11) and predictive performance (Table 12) reveals a clear evolutionary trajectory in model development, characterized by increasing sophistication in handling complex factor interactions and a demonstrable trade-off between model complexity and predictive accuracy.

Early modeling efforts focused on establishing parsimonious relationships between TOT and a limited set of factors, primarily treating variables as independent contributors. For instance, studies by Wu et al. [58] and Berghöfer et al. [16] utilized multiple regression models based on visual characteristics or driver state variables. While providing interpretable insights into individual factor effects, these models achieved moderate predictive accuracy, with goodness-of-fit metrics ranging from R^2^ = 0.40 [58] to Adjusted R^2^ = 0.182 [16]. This performance level reflects their fundamental limitation in capturing the complex, interactive nature of real-world driving environments, particularly their oversight of critical contextual factors like NDRTs and individual differences.

A significant advancement occurred with the systematic incorporation of contextual factors such as NDRT attributes [14], traffic density, and TB [100]. Methodologically, this period marked a critical shift toward addressing factor interdependencies rather than just individual effects. The adoption of VIF for diagnosing multicollinearity [14,100] and the application of linear mixed-effects models to account for data hierarchy [4] substantially improved the realism of factor integration. This methodological evolution corresponded with a measurable improvement in predictive performance, as evidenced by the generalized non-linear model [100] achieving R^2^ = 0.43, representing a meaningful advance over earlier approaches.

The most sophisticated approaches demonstrate a substantial leap in both methodological capability and predictive performance. Models capable of handling non-linear relationships and high-dimensional data, such as the Generalized Additive Model (GAM) by Li et al. [95] and the GLMM-GMM hybrid by Wang et al. [41], achieve superior goodness-of-fit (Adj. R^2^ > 0.84) and provide comprehensive error metrics (e.g., RMSE = 0.90 s). This performance level—approximately twice the explanatory power of early models—stems from their ability to move beyond static factor analysis toward dynamic process characterization. The rigorous validation of these models through likelihood ratio tests (*p* < 0.005) further confirms that their increased complexity yields statistically significant gains in predictive accuracy.

The performance metrics in Table 12 clearly demonstrate a complexity-accuracy trade-off. While simpler models offer high interpretability, their predictive power is fundamentally limited (Adj. R^2^ ≤ 0.40). In contrast, sophisticated models achieve superior accuracy (Adj. R^2^ up to 0.846) but require greater computational resources and more complex validation approaches. This trade-off directly informs model selection: researchers prioritizing causal inference for factor identification may find simpler models adequate, while those requiring high-fidelity prediction for safety-critical applications would benefit from advanced approaches despite their complexity. This systematic performance comparison establishes a foundation for evaluating these interpretable models against the high-capacity, data-driven approaches discussed subsequently.

### 5.2. Machine Learning Models

In TOT prediction research, machine learning models are widely applied due to their strong predictive capabilities and ability to handle complex data patterns. A comparative analysis of methodological approaches (Table 13) and predictive performance (Table 14) reveals distinct evolutionary trends in model complexity, feature engineering sophistication, and performance outcomes across different algorithmic paradigms.

Early machine learning applications focused on classical algorithms with carefully designed feature sets. Ref. [89] developed SVM models using eye-tracking and posture features selected through MANOVA, achieving misclassification rates of 37.7% (online) and 22.5% (offline). While effective for classification tasks, these models demonstrated limitations in handling physiological data complexity. Subsequent work by [56] employed Random Forests with permutation importance ranking, systematically incorporating physiological (heart rate, skin conductance) and environmental (scene type, traffic density) features. Their approach achieved 84.3% accuracy in classifying take-over quality, but treated TOT prediction as a classification rather than regression problem, potentially limiting precise temporal forecasting.

The field evolved toward more sophisticated ensemble methods and deep learning architectures capable of handling richer feature sets. Ref. [63]’s DeepTake model addressed class imbalance through SMOTE and feature stability via LASSO, achieving remarkable 92.8% accuracy in three-class TOT classification. Concurrently, ref. [90]’s LSTM approach demonstrated exceptional capability in modeling temporal sequences of driving conditions and driver states, achieving MAE of 0.9144 s for TOT prediction with additional precision in modeling specific action latencies (eyes: 0.2497 s, foot: 0.4650 s, hands: 0.8055 s). This represented a significant advancement from classification to precise regression-based temporal forecasting.

Recent research has emphasized regression accuracy and model interpretability. Ref. [59]’s Extra Trees Regressor, leveraging 150 s of psychophysiological data with PCA dimensionality reduction, achieved MSE of 1.6906—a 42.26% improvement over baseline models. The concurrent emergence of explainable AI approaches is exemplified by [101]’s XGBoost model with SHAP analysis, which achieved superior regression performance (MAE: 0.1507 s, RMSE: 0.2763 s, Adj. R^2^: 0.7746) while providing insights into feature importance. The most recent architectures like [99]’s ACTNet demonstrate the integration of multimodal data fusion, combining CNN-processed heatmaps with tabular features to achieve balanced performance (MAE: 1.25 s, R^2^: 0.62) with inherent interpretability through its dual-input design.

Early specialized approaches demonstrated the potential of physiological signals for TOT prediction. Ref. [91] pioneered EEG-based prediction using Bayesian Ridge Regression and Artificial Neural Networks, focusing exclusively on spectral features extracted from alpha and theta bands in the 2 s preceding an event. Their model, validated through leave-one-subject-out cross-validation, achieved impressive temporal precision with MAE ranging 0.51–0.54 s. Meanwhile, ref. [98] addressed individual differences through their M5’ nonlinear regression tree approach, incorporating 41 factors spanning demographics, driving attributes, and take-over characteristics. This hybrid regression/classification model achieved 88.59% accuracy (85.41% lateral acceleration) with RRSE of 43.57% by constructing optimal linear models for subsets partitioned based on braking/steering time requirements.

Cross-model performance analysis reveals clear algorithmic strengths and limitations. For classification tasks, DeepTake achieves the highest accuracy (92.8%) and AUC (0.96), while for regression, XGBoost demonstrates the best precision (MAE: 0.15 s). The progression from traditional SVM (MR: 37.7%) to modern architectures shows approximately 60% improvement in classification accuracy and 80% reduction in regression error. This performance gain comes with increased complexity: traditional models offer simplicity and computational efficiency, while advanced methods provide superior accuracy at the cost of greater computational demands and data requirements. The methodological evolution also shows a clear shift from basic feature selection (MANOVA) to sophisticated handling of temporal dependencies (LSTM), multimodal fusion (ACTNet), and model interpretability (SHAP), addressing different aspects of the transparency-accuracy trade-off inherent in ML approaches for safety-critical applications.

### 5.3. Cognitive Architectures Models

In TOT prediction research, cognitive architecture models provide a unique paradigm that complements data-driven approaches by explicitly simulating the multivariate relationships and complex cognitive processes underlying take-over behavior. Unlike machine learning models that prioritize predictive accuracy, these architectures emphasize mechanistic interpretability, mapping specific cognitive components (perception, decision-making, motor response) to observable TOT outcomes. The methodological approaches (Table 15) and predictive performance (Table 16) reveal distinct trade-offs between theoretical completeness, computational complexity, and empirical accuracy across different architectures.

The QN-MHP framework has been particularly productive in modeling auditory take-over cues. Ref. [49] demonstrated exceptional explanatory power (R^2^ = 0.997, RMSE = 0.148 s) by incorporating acoustic characteristics of auditory TORs within this architecture. However, this high fit comes with limited generalizability, as the model specifically optimized sound alert parameters without considering individual differences. Subsequent QN-MHP extensions addressed broader cognitive dimensions: [25] incorporated emotional states and sound cues, achieving moderate explanatory power (R^2^ = 0.4997–0.6892), while [90] integrated visual redirection, task priority, situational awareness, and trust, showing method-dependent performance variation (R^2^ = 0.76–0.97, RMSE = 3.02–8.10 s). This progression illustrates the architecture’s flexibility in incorporating additional cognitive variables, though with increasing computational complexity.

The ACT-R architecture has excelled in modeling complex psychosocial interactions. Ref. [63] established robust structural relationships between trust, system characteristics, and individual differences, achieving excellent model fit indices ($\chi^{2}$/df = 1.684, CFI = 0.948, RMSEA = 0.071). More recently, ref. [99]’s ACT-R implementation demonstrated remarkable dual-aspect predictive power, with R^2^ values of 0.9669 for take-over response time and 0.9705 for mental workload quantification, though its applicability to elderly drivers remains unverified.

The QN-ACTR architecture represents the most integrated approach, combining production-rule-based single-task models through multi-task scheduling. Ref. [103] achieved outstanding predictive accuracy (R^2^ = 0.96, RMSE = 0.5 s, MAPE = 9%) by simultaneously modeling road/traffic situations with driver attention/fatigue states. This architecture’s strength lies in its ability to handle multiple concurrent tasks, though it currently employs a single strategic approach that may not capture individual behavioral variations.

Comparative analysis reveals fundamental trade-offs between architectural paradigms. QN-MHP models excel in sensory-cognitive mapping but show variable performance when scaling complexity (R^2^ range: 0.50–0.99). ACT-R architectures demonstrate superior theoretical completeness through validated structural equations but require extensive parameterization. QN-ACTR achieves the best balance between accuracy and mechanistic interpretability but lacks flexibility in capturing individual differences. Across all architectures, there remains a noticeable gap in incorporating dynamic environmental factors and system characteristics, with most models prioritizing cognitive variables over contextual elements.

The progression from specialized auditory models to integrated cognitive architectures reflects increasing sophistication in handling multivariate interactions. However, significant challenges persist in reconciling theoretical completeness with practical predictive power. Future developments must address the integration of individual differences, environmental dynamics, and system characteristics within computationally feasible frameworks that maintain the interpretability advantages of cognitive architectures while achieving the predictive accuracy of data-driven approaches.

### 5.4. Comparative Analysis Across Modeling Paradigms

The systematic examination of classical statistical, machine learning, and cognitive architecture models reveals fundamental trade-offs in predictive accuracy, computational efficiency, interpretability, and practical implementation constraints. This comparative analysis synthesizes evidence from Table 11, Table 12, Table 13, Table 14, Table 15 and Table 16 to establish a comprehensive framework for model selection based on specific application requirements and contextual constraints.

Machine learning models demonstrate superior predictive accuracy but at significant computational costs. As shown in Table 14, DeepTake achieves exceptional classification performance (92.8% accuracy, AUC: 0.96), representing an approximately 60% improvement over traditional SVM approaches. Similarly, XGBoost attains remarkable regression precision (MAE: 0.1507 s, Adj. R^2^: 0.7746), outperforming classical statistical models by substantial margins. However, this accuracy advantage comes with substantial computational demands—training complex architectures like LSTM or ACTNet requires extensive data preprocessing, specialized hardware, and significant inference times that may challenge real-time deployment in embedded vehicle systems. The feature engineering complexity evident in Table 13, particularly for multimodal approaches like ACTNet, further compounds these computational requirements.

In contrast, classical statistical models offer compelling advantages in computational efficiency and transparency. As summarized in Table 12, the Generalized Additive Model achieves respectable performance (training Adj. R^2^: 0.747, test MAE: 0.72 s) with minimal computational overhead, while providing full interpretability through explicit parameter estimates and significance testing. The methodological transparency shown in Table 11, featuring rigorous collinearity checks and feature selection procedures, makes statistical models particularly valuable for safety-critical applications where regulatory compliance and diagnostic capability are prioritized over maximal accuracy. This paradigm demonstrates robust performance with relatively small datasets, avoiding the data hunger that characterizes many machine learning approaches.

Cognitive architecture models occupy a unique position in the accuracy-interpretability spectrum. While QN-ACTR demonstrates competitive predictive performance (R^2^: 0.96, RMSE: 0.5 s) as shown in Table 16, their primary value lies in mechanistic explanatory power. These models explicitly simulate cognitive processes—from auditory perception in QN-MHP models to decision-making in ACT-R architectures—providing testable theoretical frameworks that transcend purely predictive functions. The methodological approaches detailed in Table 15 highlight their strength in modeling complex multivariate relationships, making them particularly valuable for designing human–machine interfaces and understanding failure modes, though their implementation complexity and parameterization requirements limit practical deployment.

The paradigms exhibit dramatically different data dependencies and generalizability characteristics. Machine learning models like Extra Trees Regressor and DeepTake require large, high-quality datasets (150 s psychophysiological data; multimodal feature sets) for effective training, creating significant data acquisition barriers. Statistical models demonstrate greater robustness with smaller samples but struggle with high-dimensional interactions, as evidenced by the moderate R^2^ values (typically 0.40–0.45) in earlier studies. Cognitive architectures, while data-efficient for theoretical validation, require extensive domain knowledge for parameterization and may lack generalizability across diverse populations and scenarios, particularly for models that neglect individual differences as noted in several QN-MHP implementations.

Real-world deployment introduces additional practical constraints. The computational intensity of advanced machine learning models (LSTM inference latency, XGBoost memory requirements) may challenge embedded system limitations in actual vehicles. Statistical models offer lightweight implementation but with accuracy ceilings that may be insufficient for highly dynamic scenarios. Cognitive architectures face fundamental challenges in real-time simulation speeds, though their component-based nature permits selective implementation of validated submodules for specific applications.

This comprehensive analysis suggests a complementary rather than competitive relationship between modeling paradigms. The progression from simple statistical models to sophisticated machine learning and cognitive architectures represents an evolution in handling complexity rather than a linear hierarchy of superiority. Hybrid approaches—such as using cognitive architectures to inform feature engineering for machine learning models, or employing statistical models for rapid prototyping—may optimize the accuracy-interpretability-efficiency trade-off. Future research should develop context-aware selection frameworks that match model capabilities to specific application requirements, whether prioritizing interpretability for regulatory approval, accuracy for safety-critical functions, or mechanistic insight for interface design.

## 6. Discussion

### 6.1. Experimental Limitations

While this review synthesizes significant advancements in TOT prediction, the methodological foundations of the field face substantial challenges that threaten the validity and generalizability of research findings. These limitations span data collection methodologies, experimental designs, and technological implementations, collectively constraining the translation of laboratory insights to real-world applications.

The ecological validity gap in simulation methodologies represents a fundamental concern. The heavy reliance on simulator-based studies introduces questions about their ability to capture real-world driving conditions. Desktop and cockpit simulators, while offering practical advantages in safety and scenario control, fail to replicate the multisensory feedback and psychological pressures of actual driving. The absence of genuine risk perception, gravitational forces, and real-world consequences likely leads to attenuated physiological responses and altered behavioral patterns. The Wizard of Oz approach, though cost-effective, introduces additional artificiality through human-operated automation that cannot precisely replicate the timing and behavior patterns of true automated systems. These methodological compromises cast uncertainty on whether reported TOT values accurately reflect human performance in genuine driving emergencies.

Data quality and sensor reliability concerns present significant challenges for the field. The sophisticated multi-modal data acquisition frameworks face implementation issues that are frequently underreported. Eye-tracking systems, while prevalent in TOT research, are notoriously susceptible to calibration drift, particularly during extended experimental sessions. Physiological sensors encounter signal quality issues from movement artifacts, electrode slippage, and individual physiological differences that standard normalization techniques may not fully address. The critical challenge of temporal synchronization across multiple data streams introduces millisecond-level uncertainties that can significantly impact the precise temporal measurements essential for TOT analysis. Furthermore, the dependency on post-hoc data cleaning and interpolation methods to handle missing values raises questions about dataset integrity.

Sample diversity and representativeness issues limit the generalizability of findings. Current research populations demonstrate significant limitations in demographic and psychological diversity. Most studies recruit from convenience samples, neglecting critical populations such as elderly drivers, individuals with disabilities, or those with limited technological experience. The psychological state control problem represents another fundamental challenge—while studies attempt to induce or measure states like fatigue, stress, or distraction, these manipulations often create artificial conditions that may not reflect naturalistic states. Furthermore, the limited sample sizes common in simulator studies provide insufficient statistical power for detecting subtle but potentially important effects.

Technological generalizability constraints emerge from the rapid evolution of automated vehicle technologies. Experimental systems often implement simplified automation behaviors that may not represent current industry capabilities, while TOR designs frequently lack the sophistication of production systems. The artificial simplification of driving scenarios fails to capture the complexity of real-world driving environments. Additionally, the static nature of most experimental designs—using fixed time budgets and uniform TOR modalities—does not account for the adaptive systems that will characterize future automated vehicles.

Methodological standardization deficits present barriers to cross-study comparisons and validation. The field suffers from a lack of standardized metrics, protocols, and validation frameworks. Variations in TOT definitions complicate cross-study comparisons, while diverse data processing pipelines introduce additional variability. The almost universal focus on short-term take-over performance neglects the extended adaptation period that may be critical for understanding complete take-over quality. Furthermore, the validation approaches predominantly used may produce overly optimistic performance estimates compared to real-world deployment conditions.

These collective limitations necessitate a more critical interpretation of existing TOT findings and highlight the importance of methodological transparency in future research. While simulation studies provide valuable initial insights, their constraints underscore the need for complementary naturalistic studies and more sophisticated validation approaches that better bridge the gap between laboratory conditions and real-world driving environments. Future work should address these limitations through improved experimental designs, enhanced sensor reliability, more diverse participant sampling, and the development of standardized evaluation frameworks.

### 6.2. Model Limitations

While the comparative analysis reveals distinct advantages across modeling paradigms, each approach faces significant practical constraints that impact real-world deployment. These limitations extend beyond predictive accuracy to encompass computational efficiency, implementation feasibility, and operational constraints that are critical for safety-critical automotive applications.

Classical statistical models demonstrate excellent computational efficiency and transparency but face fundamental constraints in real-time prediction scenarios. Their lightweight nature enables rapid execution on resource-constrained embedded systems, with inference times typically measured in milliseconds. However, this efficiency comes at the cost of limited capacity to handle complex, high-dimensional interactions between factors. The sequential nature of feature extraction pipelines in statistical approaches introduces additional latency concerns, particularly when processing real-time sensor data streams. More critically, the extensive preprocessing requirements for data normalization and assumption validation create implementation bottlenecks that may compromise their utility in dynamic driving environments where conditions change rapidly.

Machine learning models achieve remarkable predictive performance but present substantial deployment challenges due to their computational intensity. The training phase for architectures like DeepTake and XGBoost demands significant computational resources, specialized hardware, and extended processing times that are impractical for frequent model updates in vehicle systems. During inference, while tree-based models like XGBoost demonstrate reasonable efficiency, deep learning approaches such as LSTM networks introduce considerable latency due to their sequential processing nature. The feature extraction overhead for multimodal approaches like ACTNet further compounds these timing constraints, creating potential bottlenecks for real-time TOT prediction where millisecond-level responses are critical for safety. Additionally, the substantial memory footprint of these models challenges the storage limitations of automotive-grade computing systems.

Cognitive architecture models offer unparalleled mechanistic insight but face fundamental scalability limitations. The computational overhead of simulating complex cognitive processes in architectures like ACT-R and QN-MHP results in inference times that are orders of magnitude slower than statistical or machine learning approaches. This makes real-time deployment currently infeasible for comprehensive cognitive models, restricting their practical application to offline analysis and system design. The parameterization complexity of these architectures requires extensive domain expertise and creates significant barriers for adaptation to new driving scenarios or population demographics. Furthermore, the integration of multiple cognitive modules introduces cumulative latency that exacerbates timing constraints in time-critical take-over situations.

Across all paradigms, the feature engineering pipeline presents universal timing challenges that are frequently overlooked in performance reporting. The processes of sensor data acquisition, signal preprocessing, feature extraction, and temporal alignment introduce substantial latency that can exceed model inference times themselves. For physiological features such as EEG and EOG signals, the computational overhead of noise filtering, artifact removal, and spectral analysis creates additional delays that impact the temporal precision of TOT predictions. This is particularly problematic for time-sensitive applications where prediction latency directly affects system responsiveness and safety margins.

The trade-offs between model complexity, prediction accuracy, and computational feasibility reveal fundamental constraints in current approaches. While machine learning models achieve superior accuracy, their computational demands render them problematic for resource-constrained automotive systems. Statistical models offer implementation efficiency but lack the sophistication for complex scenario handling. Cognitive architectures provide theoretical completeness but face impractical computational requirements. This trilemma underscores the need for context-aware model selection that balances theoretical sophistication with practical implementation constraints, particularly for safety-critical applications where both accuracy and timing reliability are paramount.

Future research directions should prioritize the development of adaptive complexity models that can dynamically adjust their computational demands based on scenario criticality and available resources. Hybrid approaches that combine the efficiency of statistical models for baseline performance with the power of machine learning for complex scenarios offer promising pathways. Additionally, greater emphasis on computational efficiency metrics alongside accuracy measures would provide more realistic assessments of deployment feasibility. The field must also address the significant gap in standardized benchmarking for real-time performance, including end-to-end latency measurements from sensor input to prediction output, to better evaluate practical utility beyond theoretical capabilities.

## 7. Future Directions

Building upon the systematic analysis of current limitations in TOT prediction research, future work should pursue an integrated advancement across methodological, technical, and practical dimensions. The translation of theoretical models into real-world applications necessitates a concerted focus on ecological validity, computational efficiency, and scalable implementation frameworks.

Future experimental methodologies must bridge the simulation-reality gap through hybrid validation approaches that combine rigorous controlled studies with naturalistic observations. This requires developing dynamic scenario generation systems capable of adapting complexity based on real-time driver performance assessment. Significant investments should be directed toward creating large-scale, diverse datasets through collaborative initiatives that employ federated learning techniques while ensuring participant privacy. Particular attention must be paid to representative sampling strategies that adequately include critical populations such as elderly drivers and individuals with varying levels of technological proficiency. Concurrently, the field needs standardized benchmark suites that simulate real-world constraints, including standardized testing protocols across diverse operational domains and failure mode analyses specific to prediction errors.

Model development should prioritize hybrid architectures that intelligently leverage the complementary strengths of different paradigms. A promising direction involves hierarchical systems where lightweight statistical models provide real-time baseline predictions, while more sophisticated machine learning or cognitive models activate selectively based on scenario criticality. Such systems would benefit from reinforcement learning frameworks for dynamic model selection, optimizing the accuracy-efficiency trade-off according to contextual factors like time pressure and driver state. To address computational constraints, research should explore model compression techniques tailored for automotive systems and temporal-efficient architectures that improve latency-accuracy trade-offs. The development of incremental learning capabilities will be crucial for personalization without requiring complete model retraining.

The explainability challenge demands integrated solutions that combine technical explainable AI methods with domain-specific cognitive principles. Future work should establish unified explanation frameworks that produce actionable insights for system designers and regulators alike. This must be coupled with comprehensive evaluation metrics that assess not only predictive performance but also computational efficiency, robustness under sensor failure, and explanation fidelity under realistic automotive constraints.

For practical implementation, the field requires certification frameworks specifically designed for safety-critical prediction systems. This includes standardized validation methodologies, reference implementations for automotive-grade systems, and clear accountability frameworks for system limitations. Industry-academia partnerships should document implementation challenges and solutions for computational constraints, real-time requirements, and safety certification processes. Long-term research vision should focus on developing truly adaptive take-over systems that dynamically adjust intervention strategies based on continuous assessment of driver state, environmental complexity, and system performance.

## 8. Conclusions

This comprehensive review has systematically examined the methodological approaches, predictive capabilities, and practical limitations in TOT prediction research, focusing on three primary modeling paradigms: classical statistical models, machine learning approaches, and cognitive architectures. The analysis demonstrates that while significant theoretical progress has been achieved, substantial challenges remain in translating these advances into practical applications for automated driving systems.

The comparative assessment reveals distinct trade-offs between model sophistication and practical implementability. Classical statistical models provide interpretability and computational efficiency but are limited in handling complex, high-dimensional interactions. Machine learning approaches achieve superior predictive accuracy through ensemble methods and deep learning architectures, yet face substantial computational demands and interpretability challenges. Cognitive architecture models offer unique insights into driver cognitive processes but encounter fundamental barriers in real-time deployment due to their computational intensity and parameterization complexity.

The field’s progression shows a clear evolution from simple factor analysis to sophisticated temporal modeling, with machine learning approaches demonstrating marked improvement in classification accuracy and significant reduction in regression error compared to early methods. However, these performance gains introduce substantial computational complexity, creating implementation challenges for resource-constrained automotive systems. The systematic identification of limitations across experimental methodologies, data quality concerns, and model constraints highlights the critical need for more robust validation frameworks.

Future advancements should prioritize three key areas: first, developing hybrid validation protocols that combine controlled studies with naturalistic observations to enhance ecological validity; second, creating adaptive modeling frameworks that dynamically balance accuracy and efficiency based on contextual requirements; third, establishing standardized benchmarking methodologies that assess both theoretical performance and practical implementation constraints. These directions will enable the development of TOT prediction systems that meet the rigorous demands of automotive safety standards while maintaining theoretical sophistication.

The integration of these approaches through collaborative efforts across research institutions and industry partners will be essential for creating effective human–automation interaction in increasingly complex driving environments. By addressing the identified methodological and implementation challenges, the field can advance toward practical solutions that enhance both safety and user experience in real-world automated driving applications.

## Figures and Tables

**Table 1:** Top 10 Countries by Total Citation Frequency.

Countries	Total Cited Count
Germany	901
United Kingdom	461
Netherlands	345
United States	308
Australia	219
China	158
Republic of Korea	138
Austria	53
Japan	51
France	48

**Table 2:** Top 10 Countries by Average Citation Frequency.

Countries	Average Cited Count
Australia	109.50
Netherlands	57.50
United Kingdom	51.20
Germany	42.90
Austria	17.70
Morocco	13.00
Republic of Korea	12.50
United States	11.00
France	9.60
Japan	7.30

**Table 3:** Number of Publications by Institution.

Institutes	Number of Publications
University of Michigan	19
Tsinghua University	15
Beihang University	11
Delft University of Technology	11
Technical University of Berlin	11
Technical University of Munich	11
Chalmers University of Technology	9
University of Ljubljana	9
University of Southampton	8
Wuhan University of Technology	8

**Table 4:** Number of Publications Included in Each Journal.

Journal	Number of Publications
Transportation Research Part F: Traffic Psychology and Behaviour	21
Accident Analysis and Prevention	18
Human Factors	15
IEEE Transactions on Intelligent Transportation Systems	9
Applied Ergonomics	6
IEEE Access	5
IEEE Transactions on Human–Machine Systems	4
Transportation Research Record	4
Applied Sciences-Basel	3
International Journal of Human-Computer Interaction	3

**Table 7:** Take-over Modes in Autonomous Vehicle Experiments.

Take-Over Mode	Specific Content	Ref.
Steering	Turn the steering wheel by a certain angle	[54,55]
Pedal	Press brake pedal percentage	[30]
Button	Fixed button on: Display/Steering wheel/Gear position	[41,49,56,57]
Pedal or Steering		[31,32,48,51,58,59,60]
Pedal/Steering/Button		[22,61]
Custom Methods	Press the lever behind the steering wheel	[16]
	Touch the steering wheel and press the button	[62]
	Press two buttons on the steering wheel simultaneously	[63]

**Table 8:** Typical Take-over Events in Take-over Experiments.

Event Type	Specific Content	Ref.
System Longitudinal Function Limited	Obstacle ahead	[40,55,59,69]
	Steep slope	[59]
	Vehicle ahead stationary	[22,26,70]
	Construction site	[22,23,40,48]
	Vehicle ahead braking	[15,23]
	Obstacle during lane change of front vehicle	[58]
	Pedestrian or animal intrusion	[26,59,71]
	Sudden vehicle entry	[15,22,23]
	Overtaking	[23,42]
	Rainy day	[59,71]
	Foggy day	[23,71,72]
System Lateral Function Limited	Blurred lane markings	[15,59]
	Ramp entrance/exit	[17,40,67,72,73]
System Failure	Partial system function failure	[71,74,75,76,77]

**Table 9:** Typical Experimental Equipment in Take-over Experiments.

Experimental Equipment	Ref.
Desktop Simulator	[8,31,34,47,79]
Cockpit Simulator	[25,30,32,33,35,80,81,82,83]
Real Vehicle	[23,51,68,84,85]

**Table 11:** Methodological Overview of Classical Statistical Models in Take-over Analysis.

Statistical Model	Independent Variables	Year	Key Methodology	Ref.
Generalized Non-linear Model	TB, traffic density, NDRTs, task repetitiveness, lane, driver age	2018	Diagnosis via VIF Significant predictors (*p* < 0.05)	[100]
Multiple Regression Model	Visual behavior, drowsiness, attitude, ACC experience, reaction speed, age, gender	2018	Significant predictors (*p* < 0.05)	[16]
linear mixed-effects model	Event urgency, device usage, visual NDRTs, TOR type, driver experience	2019	Within-study: Condition-wise TOT differences (Wilcoxon) Between-study: TOT correlations with study variables (Pearson/Spearman)	[4]
Multiple Linear Regression model	Physical/visual/cognitive NDRTs	2021	Feature selection: Backward elimination (*p* < 0.05) Collinearity check: VIF values 1.35–3.51, below critical threshold (VIF > 10)	[14]
Multiple Regression Model	Visual characteristics	2021	Preliminary analysis: Pearson correlations between eye-movement measures and RT Model building: Stepwise regression with backward elimination (*p* < 0.05)	[58]
Generalized Additive Model	Fatigue, traffic situations, TB	2022	Validation: Spearman correlation (POF, MSRD, TTBT) Data: 357 take-overs, train/test split (286/71) VIF: Low values, no multicollinearity	[95]
Generalized Linear Mixed Model	Preceding speed, autonomy duration, TB, trajectory, behavior	2024	Feature selection: EMD-based screening for optimal GMM variable combination Driving state classification: GMM to detect unstable-stable transitions Model validation: GLMM compared to GLM via likelihood ratio test	[41]

**Table 12:** Predictive Performance of Classical Statistical Models in Take-over Analysis.

Model	Goodness-of-Fit	Error Metrics	Statistical Significance	Ref.
Generalized Non-linear Model	R^2^ = 0.43	RMSE = 0.81 s	–	[100]
Multiple Regression Model	Adjusted R^2^ = 0.182	–	F(9,148)=4.669, *p* < 0.001	[16]
Linear Mixed-effects Model	–	–	Most predictors: p<0.05	[4]
Multiple Linear Regression	MRT: R^2^ = 0.326 (Adj. R^2^ = 0.313)	–	Validation correlation:	[14]
(Component Models)	PARST: R^2^ = 0.304 (Adj. R^2^ = 0.274)		r=0.243 (individual),	
	GT: R^2^ = 0.373 (Adj. R^2^ = 0.364)		r=0.931 (mean by NDRT)	
Multiple Regression Model	R^2^ = 0.40	–	*F*-statistic, p< 0.001	[58]
Generalized Additive Model	Training Adj. R^2^ = 0.747	Test Set: MAE = 0.72 s, RMSE = 0.90 s	–	[95]
		Adaptive Strategy: MAE = 0.71 s, RMSE = 0.86 s		
Mixed Model	Critical Scenario: Adj. R^2^ = 0.839	–	Likelihood Ratio test:	[41]
(GMM & GLMM)	Non-critical Scenario: Adj. R^2^ = 0.846		GLMM > GLM (*p* < 0.005)	

**Table 13:** Methodological Overview of Machine Learning Models in Take-over Analysis.

Model	Features	Year	Key Methodology	Ref.
SVM	Eye movements, posture	2019	Feature selection: MANOVA	[89]
RF	Heart rate, skin conductance, eye tracking, scene type, traffic density	2020	Method: Random Forest permutation importance ranking Process: Sequential addition of top-ranked features	[56]
DeepTake	Visual features, skin conductance, heart rate	2021	SMOTE class imbalance, LASSO stable selection, Random Forest importance ranking	[63]
LSTM	Driving conditions, driver state, distractions, control transfer timing	2021	Ablation studies on feature combinations	[90]
Extra Trees	150 s psychophysiological data	2021	Variance Threshold, PCA	[59]
Bayesian Ridge + ANN	EEG spectral features	2022	Validation: leave-one-subject-out cross-validation	[91]
M5’ nonlinear regression tree	41 factors (demographics, driving attributes, take-over characteristics)	2023	The dataset is divided according to rules such as “the time required for the first braking/steering”, and an optimal linear model is constructed for each subset.	[98]
ACTNet	Driver state, demographics, traffic situations, interaction features	2024	Dual-input ACTNet fusing CNN-processed heatmaps and tabular features	[99]
XGBoost	Personal traits, environment, situational awareness	2024	Model Interpretation: SHAP analysis for global/local explanations. Ablation analysis via Base Model (BM) vs. enhanced model (BM+SA)	[101]

**Table 14:** Predictive Performance of Machine Learning Models in Take-over Analysis.

Model	Primary Task	Classification Metrics	Regression Error Metrics	Goodness-of-Fit	Ref.
SVM	Classification (Online vs. Offline)	Online MR: 38.7% Offline MR: 22.5% With Posture: 37.7%	–	–	[89]
RF	Classification (Good/Bad take-over)	Accuracy: 84.3% F1: 64.0% Precision: 64.5% Recall: 63.9%	–	–	[56]
DeepTake	Classification (3-class: TOT Level)	Accuracy: 92.8% Weighted F1: 0.87 AUC: 0.96	–	–	[63]
LSTM	Regression (Multiple Targets)	–	TOT MAE: 0.9144 s Eyes MAE: 0.2497 s Foot MAE: 0.4650 s Hands MAE: 0.8055 s	–	[90]
Extra Trees	Regression	–	RT MSE: 1.6906 MaxSWA MSE: 161.93	–	[59]
Bayesian Ridge + ANN	Regression	–	Best MAE: 0.51–0.54 s (Alpha/Theta bands)	–	[91]
M5’	Mixed (Regression & Classification)	Acc: 88.59%	Reaction Time: 43.57% Lat. Accel: 85.41%	–	[98]
ACTNet	Regression	–	MAE: 1.25 ± 0.21 s RMSE: 1.60 ± 0.20 s	R^2^: 0.62 ± 0.04	[99]
XGBoost	Regression	–	MAE: 0.1507 s RMSE: 0.2763 s	Adj. R^2^: 0.7746	[101]

**Table 15:** Methodological Overview of Cognitive Architecture Models in Take-over Analysis.

Model	Predictors	Year	Key Methodology	Ref.
QN-ACTR	Road/traffic situations, driver attention/fatigue	2019	Modeling:Production-rule-based single-task models integrated via QN-ACTR’s multi-task scheduling	[20]
QN-MHP	Emotional states, sound cue frequency/repetition	2020	Statistical tests were chosen based on normality of residuals: parametric tests (e.g., ANOVA) for normal data, non-parametric tests (e.g., Mann-Whitney U) otherwise.	[32]
QN-MHP	Sound characteristics (loudness/semantics/acoustics)	2021	Statistical Analysis: Repeated measures ANOVA with Bonferroni correction for multiple comparisons	[49]
ACT-R	Trust, system/environment characteristics, individual differences	2021	Validated the measurement model using Confirmatory Factor Analysis, followed by path analysis to test the structural relationships	[102]
QN-MHP	Visual redirection, task priority, situational awareness, trust	2022	Modeling the decision-making mechanism through Markov chains to simulate real-time transitions between monitoring, NDRTs, and take-over	[73]
ACT-R	Psycho-load in take-over scenarios	2024	Quantifying workload via ACT-R module activation/decay; Simulating adaptive decision-making between take-over and NDRTs	[55]

**Table 16:** Predictive Performance of Cognitive Architecture Models in Take-over Analysis.

Model	Goodness-of-Fit (R^2^)	Error Metrics	Model Fit Indices	Ref.
QN-ACTR	R^2^ = 0.96	RMSE = 0.5 s MAPE = 9%	-	[20]
QN-MHP	All data: R^2^ = 0.4997 Excl. 8-rep/s warnings: R^2^ = 0.6892	-	-	[32]
QN-MHP	R^2^ = 0.997	RMSE = 0.148 s	-	[49]
ACT-R	-	-	$\chi^{2}$/df = 1.684 (<3) CFI = 0.948 (>0.9) RMSEA = 0.071 (<0.08) GFI = 0.901 (>0.9)	[102]
QN-MHP	Method 1 (by driver): R^2^ = 0.76 Method 2 (by event): R^2^ = 0.97	RMSE = 8.10 s RMSE = 3.02 s	-	[73]
ACT-R	take-over Response Time: R^2^ = 0.9669 Mental Workload: R^2^ = 0.9705	-	-	[55]

## References

[1] Gangadharaiah R., Mims L., Jia Y., Brooks J. (2024). Opinions from Users Across the Lifespan about Fully Autonomous and Rideshare Vehicles with Associated Features. SAE Int. J. Adv. Curr. Prac. Mobil..

[2] Mohammed K., Abdelhafid M., Kamal K., Ismail N., Ilias A. (2023). Intelligent driver monitoring system: An Internet of Things-based system for tracking and identifying the driving behavior. Comput. Stand. Interfaces.

[3] Cigno R.L., Segata M. (2022). Cooperative driving: A comprehensive perspective, the role of communications, and its potential development. Comput. Commun..

[4] Zhang B., De Winter J., Varotto S., Happee R., Martens M. (2019). Determinants of take-over time from automated driving: A meta-analysis of 129 studies. Transp. Res. Part F Traffic Psychol. Behav..

[5] Wang W., Li Q., Zeng C., Li G., Zhang J., Li S., Cheng B. (2023). Review of Take-over Performance of Autonomous Driving: Influencing Factors, Models, and Evaluation Methods. China J. Highw. Transp..

[6] Wang C., Ren W., Xu C., Zheng N., Peng C., Tong H. (2025). Exploring the Impact of Conditionally Automated Driving Vehicles Transferring Control to Human Drivers on the Stability of Heterogeneous Traffic Flow. IEEE Trans. Intell. Veh..

[7] Aria M., Cuccurullo C. (2017). bibliometrix: An R-tool for comprehensive science mapping analysis. J. Informetr..

[8] Minhas S., Hernández-Sabaté A., Ehsan S., McDonald-Maier K.D. (2020). Effects of non-driving related tasks during self-driving mode. IEEE Trans. Intell. Transp. Syst..

[9] Rangesh A., Deo N., Greer R., Gunaratne P., Trivedi M.M. (2021). Predicting take-over time for autonomous driving with real-world data: Robust data augmentation, models, and evaluation. arXiv.

[10] Ban G., Park W. (2024). Effects of in-vehicle touchscreen location on driver task performance, eye gaze behavior, and workload during conditionally automated driving: Nondriving-related task and take-over. Hum. Factors.

[11] Müller A.L., Fernandes-Estrela N., Hetfleisch R., Zecha L., Abendroth B. (2021). Effects of non-driving related tasks on mental workload and take-over times during conditional automated driving. Eur. Transp. Res. Rev..

[12] Merlhiot G., Bueno M. (2022). How drowsiness and distraction can interfere with take-over performance: A systematic and meta-analysis review. Accid. Anal. Prev..

[13] Pan H., He H., Wang Y., Cheng Y., Dai Z. (2023). The impact of non-driving related tasks on the development of driver sleepiness and takeover performances in prolonged automated driving. J. Saf. Res..

[14] Yoon S.H., Lee S.C., Ji Y.G. (2021). Modeling takeover time based on non-driving-related task attributes in highly automated driving. Appl. Ergon..

[15] Zhang Q., Esterwood C., Pradhan A.K., Tilbury D., Yang X.J., Robert L.P. (2023). The impact of modality, technology suspicion, and ndrt engagement on the effectiveness of av explanations. IEEE Access.

[16] Berghöfer F.L., Purucker C., Naujoks F., Wiedemann K., Marberger C. Prediction of take-over time demand in conditionally automated driving-results of a real world driving study. Proceedings of the Human Factors and Ergonomics Society Europe.

[17] Bai J., Sun X., Cao S., Wang Q., Wu J. (2024). Exploring the timing of disengagement from nondriving related tasks in scheduled takeovers with pre-alerts: An analysis of takeover-related measures. Hum. Factors.

[18] Li Q., Hou L., Wang Z., Wang W., Zeng C., Yuan Q., Cheng B. (2021). Drivers’ visual-distracted take-over performance model and its application on adaptive adjustment of time budget. Accid. Anal. Prev..

[19] Hwang S., Banerjee A.G., Boyle L.N. (2020). Predicting driver’s transition time to a secondary task given an in-vehicle alert. IEEE Trans. Intell. Transp. Syst..

[20] Deng C., Cao S., Wu C., Lyu N. (2019). Modeling driver take-over reaction time and emergency response time using an integrated cognitive architecture. Transp. Res. Rec..

[21] Du N., Zhou F., Tilbury D.M., Robert L.P., Yang X.J. (2024). Behavioral and physiological responses to takeovers in different scenarios during conditionally automated driving. Transp. Res. F Traffic Psychol. Behav..

[22] Yi B., Cao H., Song X., Wang J., Guo W., Huang Z. (2023). Measurement and real-time recognition of driver trust in conditionally automated vehicles: Using multimodal feature fusions network. Transp. Res. Rec..

[23] Huang C., Yang B., Nakano K. (2024). Impact of Personality on Takeover Time and Maneuvers Shortly After Takeover Request. IEEE Trans. Intell. Transp. Syst..

[24] Huang C., Yang B., Nakano K. (2023). Impact of duration of monitoring before takeover request on takeover time with insights into eye tracking data. Accid. Anal. Prev..

[25] Sanghavi H., Zhang Y., Jeon M. (2023). Exploring the influence of driver affective state and auditory display urgency on takeover performance in semi-automated vehicles: Experiment and modelling. Int. J. Hum.-Comput. Stud..

[26] Ko S., Zhang Y., Jeon M. Modeling the effects of auditory display takeover requests on drivers’ behavior in autonomous vehicles. Proceedings of the 11th International Conference on Automotive User Interfaces and Interactive Vehicular Applications: Adjunct Proceedings.

[27] Gasne C., Paire-Ficout L., Bordel S., Lafont S., Ranchet M. (2022). Takeover performance of older drivers in automated driving: A review. Transp. Res. Part F Traffic Psychol. Behav..

[28] Muslim H., Itoh M., Liang C.K., Antona-Makoshi J., Uchida N. (2021). Effects of gender, age, experience, and practice on driver reaction and acceptance of traffic jam chauffeur systems. Sci. Rep..

[29] Becker S., Brandenburg S., Thüring M. (2024). Driver-initiated take-overs during critical evasion maneuvers in automated driving. Accid. Anal. Prev..

[30] Samani A.R., Mishra S., Dey K. (2022). Assessing the effect of long-automated driving operation, repeated take-over requests, and driver’s characteristics on commercial motor vehicle drivers’ driving behavior and reaction time in highly automated vehicles. Transp. Res. Part F Traffic Psychol. Behav..

[31] Han Y., Wang T., Shi D., Ye X., Yuan Q. (2023). The Effect of Multifactor Interaction on the Quality of Human–Machine Co-Driving Vehicle Take-Over. Sustainability.

[32] Sanghavi H.K. (2020). Exploring the Influence of Anger on Takeover Performance in Semi-Automated Vehicles. Ph.D. Thesis.

[33] Rydström A., Mullaart M.S., Novakazi F., Johansson M., Eriksson A. (2023). Drivers’ performance in non-critical take-overs from an automated driving system—An on-road study. Hum. Factors.

[34] Li S., Blythe P., Zhang Y., Edwards S., Guo W., Ji Y., Goodman P., Hill G., Namdeo A. (2022). Analysing the effect of gender on the human-machine interaction in level 3 automated vehicles. Sci. Rep..

[35] Roberts S.C., Hanson W., Ebadi Y., Talreja N., Knodler M.A., Fisher D.L. (2024). Evaluation of a 3M (mistakes, mentoring, and mastery) training program for transfer of control situations in a level 2 automated driving system. Appl. Ergon..

[36] Janssen C., Praetorius L., Borst J. (2024). PREDICTOR: A tool to predict the timing of the take-over response process in semi-automated driving. Transp. Res. Interdiscip. Perspect..

[37] Niu L., Gao S., Shi J., Wu C., Wang Y., Ma S., Wang D., Yang Z., Li H. (2024). Are warnings suitable for presentation in head-up display? A meta-analysis for the effect of head-up display warning on driving performance. Transp. Res. Rec..

[38] Laakmann F., Seyffert M., Herpich T., Saupp L., Ladwig S., Kugelmeier M., Vollrath M. Benefits of Tactile Warning and Alerting of the Driver through an Active Seat Belt System. Proceedings of the 27th International Technical Conference on the Enhanced Safety of Vehicles (ESV 2023).

[39] Yun H., Yang J.H. (2020). Multimodal warning design for take-over request in conditionally automated driving. Eur. Transp. Res. Rev..

[40] Lee S., Hong J., Jeon G., Jo J., Boo S., Kim H., Jung S., Park J., Choi I., Kim S. (2023). Investigating effects of multimodal explanations using multiple In-vehicle displays for takeover request in conditionally automated driving. Transp. Res. Part F Traffic Psychol. Behav..

[41] Wang C., Xu C., Peng C., Tong H., Ren W., Jiao Y. (2024). Predicting the duration of reduced driver performance during the automated driving takeover process. J. Intell. Transp. Syst..

[42] Yang W., Wu Z., Tang J., Liang Y. (2023). Assessing the effects of modalities of takeover request, lead time of takeover request, and traffic conditions on takeover performance in conditionally automated driving. Sustainability.

[43] Zhang Y., Ma Q., Qu J., Zhou R. (2024). Effects of driving style on takeover performance during automated driving: Under the influence of warning system factors. Appl. Ergon..

[44] Diederichs F., Muthumani A., Feierle A., Galle M., Mathis L.A., Bopp-Bertenbreiter V., Widlroither H., Bengler K. (2022). Improving driver performance and experience in assisted and automated driving with visual cues in the steering wheel. IEEE Trans. Intell. Transp. Syst..

[45] Capallera M., Meteier Q., De Salis E., Widmer M., Angelini L., Carrino S., Sonderegger A., Abou Khaled O., Mugellini E. (2023). A contextual multimodal system for increasing situation awareness and takeover quality in conditionally automated driving. IEEE Access.

[46] Martinez K.D., Huang G. (2022). In-vehicle human machine interface: Investigating the effects of tactile displays on information presentation in automated vehicles. IEEE Access.

[47] Huang G., Pitts B.J. (2022). To inform or to instruct? An evaluation of meaningful vibrotactile patterns to support automated vehicle takeover performance. IEEE Trans. Hum.-Mach. Syst..

[48] Teshima T., Niitsuma M., Nishimura H. (2024). Determining the onset of driver’s preparatory action for take-over in automated driving using multimodal data. Expert Syst. Appl..

[49] Ko S., Kutchek K., Zhang Y., Jeon M. (2022). Effects of non-speech auditory cues on control transition behaviors in semi-automated vehicles: Empirical study, modeling, and validation. Int. J. Hum.-Comput. Interact..

[50] Gruden T., Tomažič S., Sodnik J., Jakus G. (2022). A user study of directional tactile and auditory user interfaces for take-over requests in conditionally automated vehicles. Accid. Anal. Prev..

[51] Shahini F., Park J., Welch K., Zahabi M. (2023). Effects of unreliable automation, non-driving related task, and takeover time budget on drivers’ takeover performance and workload. Ergonomics.

[52] Gold C., Damböck D., Lorenz L., Bengler K. “Take over!” How long does it take to get the driver back into the loop?. Proceedings of the Human Factors and Ergonomics Society Annual Meeting.

[53] Wu H., Wu C., Lyu N., Li J. (2022). Does a faster takeover necessarily mean it is better? A study on the influence of urgency and takeover-request lead time on takeover performance and safety. Accid. Anal. Prev..

[54] Griffith M., Akkem R., Maheshwari J., Seacrist T., Arbogast K.B., Graci V. (2023). The effect of a startle-based warning, age, gender, and secondary task on takeover actions in critical autonomous driving scenarios. Front. Bioeng. Biotechnol..

[55] Oh H., Yun Y., Myung R. (2024). Driver behavior and mental workload for takeover safety in automated driving: ACT-R prediction modeling approach. Traffic Inj. Prev..

[56] Du N., Zhou F., Pulver E.M., Tilbury D.M., Robert L.P., Pradhan A.K., Yang X.J. (2020). Predicting driver takeover performance in conditionally automated driving. Accid. Anal. Prev..

[57] Leitner J., Miller L., Stoll T., Baumann M. (2023). Overtake or not—A computer-based driving simulation experiment on drivers’ decisions during transitions in automated driving. Transp. Res. Part F Traffic Psychol. Behav..

[58] Wu Y., Kihara K., Takeda Y., Sato T., Akamatsu M., Kitazaki S., Nakagawa K., Yamada K., Oka H., Kameyama S. (2021). Eye movements predict driver reaction time to takeover request in automated driving: A real-vehicle study. Transp. Res. Part F Traffic Psychol. Behav..

[59] de Salis E., Meteier Q., Capallera M., Angelini L., Sonderegger A., Khaled O.A., Mugellini E., Widmer M., Carrino S. Predicting takeover quality in conditionally automated vehicles using machine learning and genetic algorithms. Proceedings of the 4th International Conference on Intelligent Human Systems Integration.

[60] DinparastDjadid A., Lee J.D., Domeyer J., Schwarz C., Brown T.L., Gunaratne P. (2021). Designing for the extremes: Modeling drivers‘ response time to take back control from automation using Bayesian quantile regression. Hum. Factors.

[61] Körber M., Weißgerber T., Kalb L., Blaschke C., Farid M. (2015). Prediction of take-over time in highly automated driving by two psychometric tests. Dyna.

[62] Dillmann J., Den Hartigh R.J.R., Kurpiers C.M., Raisch F.K., Kadrileev N., Cox R.F.A., De Waard D. (2023). Repeated conditionally automated driving on the road: How do drivers leave the loop over time?. Accid. Anal. Prev..

[63] Pakdamanian E., Sheng S., Baee S., Heo S., Kraus S., Feng L. DeepTake: Prediction of Driver Takeover Behavior using Multimodal Data. Proceedings of the ACM CHI Conference on Human Factors in Computing Systems.

[64] Heo J., Lee H., Yoon S., Kim K. (2022). Responses to take-over request in autonomous vehicles: Effects of environmental conditions and cues. IEEE Trans. Intell. Transp. Syst..

[65] Kaduk S.I., Roberts A.P.J., Stanton N.A. (2021). Driving performance, sleepiness, fatigue, and mental workload throughout the time course of semi-automated driving—Experimental data from the driving simulator. Hum. Factors Ergon. Manuf. Serv. Ind..

[66] Li S., Blythe P., Guo W., Namdeo A. (2018). Investigation of older driver’s takeover performance in highly automated vehicles in adverse weather conditions. IET Intell. Transp. Syst..

[67] Li Y., Xuan Z. (2023). Take-Over Safety Evaluation of Conditionally Automated Vehicles under Typical Highway Segments. Systems.

[68] Scharfe-Scherf M.S.L., Russwinkel N. (2021). Familiarity and complexity during a takeover in highly automated driving. Int. J. Intell. Transp. Syst. Res..

[69] Pipkorn L., Victor T., Dozza M., Tivesten E. (2021). Automation aftereffects: The influence of automation duration, test track and timings. IEEE Trans. Intell. Transp. Syst..

[70] Li Y., Xuan Z., Li X. (2023). A study on the entire take-over process-based emergency obstacle avoidance behavior. Int. J. Environ. Res. Public Health.

[71] Chen K.T., Chen H.Y.W., Bisantz A. (2023). Adding visual contextual information to continuous sonification feedback about low-reliability situations in conditionally automated driving: A driving simulator study. Transp. Res. Part F Traffic Psychol. Behav..

[72] Zhao X., Chen H., Li Z., Li H., Gong J., Fu Q. (2022). Influence characteristics of automated driving takeover behavior in different scenarios. China J. Highw. Transp..

[73] Tan X., Zhang Y. (2024). A computational cognitive model of driver response time for scheduled freeway exiting takeovers in conditionally automated vehicles. Hum. Factors.

[74] Hong S., Yue T., You Y., Lv Z., Tang X., Hu J., Yin H. (2025). A Resilience Recovery Method for Complex Traffic Network Security Based on Trend Forecasting. Int. J. Intell. Syst..

[75] Nilsson J., Strand N., Falcone P., Vinter J. Driver performance in the presence of adaptive cruise control related failures: Implications for safety analysis and fault tolerance. Proceedings of the 43rd Annual IEEE/IFIP International Conference on Dependable Systems and Networks Workshop (DSN-W).

[76] Wei R., McDonald A.D., Garcia A., Alambeigi H. (2022). Modeling driver responses to automation failures with active inference. IEEE Trans. Intell. Transp. Syst..

[77] Jung K.H., Labriola J.T., Baek H. (2023). Projecting the planned trajectory of a Level-2 automated vehicle in the windshield: Effects on human drivers’ take-over response to silent failures. Appl. Ergon..

[78] Pipkorn L., Dozza M., Tivesten E. (2024). Driver visual attention before and after take-over requests during automated driving on public roads. Hum. Factors.

[79] Riegler A., Riener A., Holzmann C. (2022). Towards personalized 3D augmented reality windshield displays in the context of automated driving. Front. Future Transp..

[80] Radlmayr J., Gold C., Lorenz L., Farid M., Bengler K. How traffic situations and non-driving related tasks affect the take-over quality in highly automated driving. Proceedings of the Human Factors and Ergonomics Society Annual Meeting.

[81] Gold C., Körber M., Lechner D., Bengler K. (2016). Taking over control from highly automated vehicles in complex traffic situations: The role of traffic density. Hum. Factors.

[82] Gold C., Berisha I., Bengler K. Utilization of drivetime–performing non-driving related tasks while driving highly automated. Proceedings of the Human Factors and Ergonomics Society Annual Meeting.

[83] Kim H., Kim W., Kim J., Lee S.J., Yoon D., Kwon O.C., Park C.H. (2023). Study on the Take-over Performance of Level 3 Autonomous Vehicles Based on Subjective Driving Tendency Questionnaires and Machine Learning Methods. ETRI J..

[84] Shi E., Bengler K. (2022). Non-driving related tasks’ effects on takeover and manual driving behavior in a real driving setting: A differentiation approach based on task switching and modality shifting. Accid. Anal. Prev..

[85] Chen R., Kusano K.D., Gabler H.C. (2015). Driver behavior during overtaking maneuvers from the 100-car naturalistic driving study. Traffic Inj. Prev..

[86] Victor T., Dozza M., Bärgman J., Boda C.N., Engström J., Flannagan C., Lee J.D., Markkula G. (2015). Analysis of Naturalistic Driving Study Data: Safer Glances, Driver Inattention, and Crash Risk.

[87] Zhu M., Wang X., Tarko A., Fang S. (2018). Modeling car-following behavior on urban expressways in Shanghai: A naturalistic driving study. Transp. Res. Part C Emerg. Technol..

[88] Pipkorn L., Tivesten E., Flannagan C., Dozza M. (2023). Driver response to take-over requests in real traffic. IEEE Trans. Hum.-Mach. Syst..

[89] Lotz A., Weissenberger S. (2019). Predicting take-over times of truck drivers in conditional autonomous driving. Advances in Human Aspects of Transportation: AHFE 2018 Advances in Intelligent Systems and Computing.

[90] Rangesh A., Deo N., Greer R., Gunaratne P., Trivedi M.M. Autonomous vehicles that alert humans to take-over controls: Modeling with real-world data. Proceedings of the IEEE International Intelligent Transportation Systems Conference (ITSC).

[91] Rahman S.U., O’Connor N., Lemley J., Healy G. Using pre-stimulus EEG to predict driver reaction time to road events. Proceedings of the 44th Annual International Conference of the IEEE Engineering in Medicine & Biology Society (EMBC).

[92] Li Q., Wang Z., Wang W., Zeng C., Wu C., Li G., Heh J.S., Cheng B. (2023). A human-centered comprehensive measure of take-over performance based on multiple objective metrics. IEEE Trans. Intell. Transp. Syst..

[93] Yi B., Cao H., Song X., Wang J., Zhao S., Guo W., Cao D. (2024). How can the trust-change direction be measured and identified during takeover transitions in conditionally automated driving? Using physiological responses and takeover-related factors. Hum. Factors.

[94] Dogan D., Bogosyan S., Acarman T. (2022). Evaluation of takeover time performance of drivers in partially autonomous vehicles using a wearable sensor. J. Sens..

[95] Li Q., Wang Z., Wang W., Zeng C., Li G., Yuan Q., Cheng B. (2021). An adaptive time budget adjustment strategy based on a take-over performance model for passive fatigue. IEEE Trans. Hum.-Mach. Syst..

[96] Araluce J., Bergasa L.M., Ocaña M., López-Guillén E., Gutiérrez-Moreno R., Arango J.F. (2022). Driver take-over behaviour study based on gaze focalization and vehicle data in CARLA simulator. Sensors.

[97] Ayoub J., Du N., Yang X.J., Zhou F. (2022). Predicting driver takeover time in conditionally automated driving. IEEE Trans. Intell. Transp. Syst..

[98] Gruden T., Jakus G. (2023). Determining key parameters with data-assisted analysis of conditionally automated driving. Appl. Sci..

[99] Liu W., Li Q., Wang W., Wang Z., Zeng C., Cheng B. (2024). Deep Learning Based Take-Over Performance Prediction and Its Application on Intelligent Vehicles. IEEE Trans. Intell. Veh..

[100] Gold C., Happee R., Bengler K. (2018). Modeling take-over performance in level 3 conditionally automated vehicles. Accid. Anal. Prev..

[101] Chen H., Zhao X., Li H., Gong J., Fu Q. (2024). Predicting driver’s takeover time based on individual characteristics, external environment, and situation awareness. Accid. Anal. Prev..

[102] Jin M., Lu G., Chen F., Shi X., Tan H., Zhai J. (2021). Modeling takeover behavior in level 3 automated driving via a structural equation model: Considering the mediating role of trust. Accid. Anal. Prev..

[103] El Jouhri A., El Sharkawy A., Paksoy H., Youssif O., He X., Kim S., Happee R. (2023). The influence of a color themed HMI on trust and take-over performance in automated vehicles. Front. Psychol..

